# A Highly Conserved *Toxo1* Haplotype Directs Resistance to Toxoplasmosis and Its Associated Caspase-1 Dependent Killing of Parasite and Host Macrophage

**DOI:** 10.1371/journal.ppat.1004005

**Published:** 2014-04-03

**Authors:** Pierre Cavailles, Pierre Flori, Olivier Papapietro, Cordelia Bisanz, Dominique Lagrange, Ludovic Pilloux, Céline Massera, Sara Cristinelli, Delphine Jublot, Olivier Bastien, Corinne Loeuillet, Delphine Aldebert, Bastien Touquet, Gilbert J. Fournié, Marie France Cesbron-Delauw

**Affiliations:** 1 UMR 5163, Centre National de la Recherche Scientifique (CNRS), Grenoble, France; 2 Université Grenoble 1, Grenoble, France; 3 GIMAP, EA 3064, Saint-Etienne, France; 4 UMR Inserm, U1043, Toulouse, France; 5 Université de Toulouse, UPS, Centre de Physiopathologie de Toulouse Purpan (CPTP), Toulouse, France; 6 UMR 5168, CNRS/INRA, Université Joseph Fourier, CEA, Grenoble, France; University of Massachusetts, United States of America

## Abstract

Natural immunity or resistance to pathogens most often relies on the genetic make-up of the host. In a LEW rat model of refractoriness to toxoplasmosis, we previously identified on chromosome 10 the *Toxo1* locus that directs toxoplasmosis outcome and controls parasite spreading by a macrophage-dependent mechanism. Now, we narrowed down *Toxo1* to a 891 kb interval containing 29 genes syntenic to human 17p13 region. Strikingly, *Toxo1* is included in a haplotype block strictly conserved among all refractory rat strains. The sequencing of *Toxo1* in nine rat strains (5 refractory and 4 susceptible) revealed resistant-restricted conserved polymorphisms displaying a distribution gradient that peaks at the bottom border of *Toxo1*, and highlighting the NOD-like receptor, *Nlrp1a*, as a major candidate. The Nlrp1 inflammasome is known to trigger, upon pathogen intracellular sensing, pyroptosis programmed-cell death involving caspase-1 activation and cleavage of IL-1β. Functional studies demonstrated that the *Toxo1*-dependent refractoriness *in vivo* correlated with both the ability of macrophages to restrict *T. gondii* growth and a *T. gondii*-induced death of intracellular parasites and its host macrophages. The parasite-induced cell death of infected macrophages bearing the LEW-*Toxo1* alleles was found to exhibit pyroptosis-like features with ROS production, the activation of caspase-1 and IL1-β secretion. The pharmacological inactivation of caspase-1 using YVAD and Z-VAD inhibitors prevented the death of both intravacuolar parasites and host non-permissive macrophages but failed to restore parasite proliferation. These findings demonstrated that the *Toxo1*-dependent response of rat macrophages to *T. gondii* infection may trigger two pathways leading to the control of parasite proliferation and the death of parasites and host macrophages. The NOD-like receptor NLRP1a/Caspase-1 pathway is the best candidate to mediate the parasite-induced cell death. These data represent new insights towards the identification of a major pathway of innate resistance to toxoplasmosis and the prediction of individual resistance.

## Author Summary

Toxoplasmosis is a ubiquitous parasitic infection causing a wide spectrum of diseases. It is usually asymptomatic but can lead to severe ocular and neurological disorders. The host factors that determine natural resistance to toxoplasmosis are yet poorly characterized. Among the animal models to study susceptibility to toxoplasmosis, rats develop like humans a subclinical chronic infection. The finding of a total resistance in the LEW rat strain has allowed genetic studies leading to the identification of *Toxo1*, a unique locus that controls the outcome of toxoplasmosis. In this report, a panel of recombinant inbred rat strains was used to genetically reduce the *Toxo1* locus, on chromosome 10, to a limited region containing 29 genes. This locus is highly conserved among five resistant, by comparison to four susceptible, rat strains, indicating that refractoriness to toxoplasmosis could be predicted. The *Toxo1*-controlled refractoriness depends on the ability of macrophages to restrict parasite proliferation and the rapid death of both *T. gondii* and host macrophages *in vitro*. The NOD-like receptor NLRP1a/Caspase-1 pathway is the best candidate to mediate the parasite-induced cell death. Our data represent new insights towards the identification of a major pathway of innate immunity that protects from toxoplasmosis.

## Introduction

*Toxoplasma gondii* is a widespread obligate intracellular protozoan parasite. One preeminent aspect of its life cycle is the establishment of a chronic infection in humans and many other vertebrate hosts [1]. Toxoplasmosis is most often asymptomatic depending on the parasite's ability to elicit host protective immunity [1]. A serious threat to human health can occur under congenital infection or reactivation of a latent infection in immunodeficient patients [2]. Epidemiological studies have indicated that the phenotypic expression of toxoplasmosis depends on the genetic make-up of both the host and the parasite [3], [4]. Variations in the outcome of *Toxoplasma* infection after exposure to similar risk factors [5], [6] and twin studies [7] support a significant role of the human host genetic background in the susceptibility to toxoplasmosis. Nevertheless, genetic studies in human are hampered by both population heterogeneity and environment variability. In experimental conditions, genetic and environmental factors are under control. Rats, like humans, usually develop subclinical toxoplasmosis. This contrasts with the severity of the disease developed in most strains of mice. Interestingly, an unexpected refractoriness to *T. gondii* infection was found in the LEW rat strain [8]. Compared to susceptible BN rats, infected LEW indeed displayed negative serology and lack of cyst burden in their brain [9]. Refractoriness of LEW rats was found to be a dominant trait dependent on hematopoietic cells [9]. It is associated with the ability of macrophages to restrict parasite proliferation *in vitro* [10].

Further genetic studies using LEW resistant and BN susceptible rats and derived reciprocal congenic strains have allowed the mapping of a locus named *Toxo1*, which fully controls the refractoriness of LEW rats to toxoplasmosis. *Toxo1* has been confined to 7.6 megabases, on rat chromosome 10 (Rn10q.24) [10]. Recently, hNlrp1 a major candidate gene present in the orthologous region to *Toxo1* in the human genome (Hs 17p32.2-p13.1) has been associated with human congenital toxoplasmosis [6].

In the present work, we used genetic dissection with a panel of BN and LEW sub-congenic rats and haplotype analysis of chromosome 10 on nine inbred rat strains either susceptible or resistant to define the localization of the gene or set of genes at work in *Toxo1* and to analyze the mechanisms of toxoplasmosis refractoriness. We were able to localize the *Toxo1* locus in a 891 kb region highly conserved in all resistant strains of rat. Sequencing of this locus in these nine strains revealed a high concentration of resistant-restricted conserved mutations at the bottom border of *Toxo1* around *Nlrp1*. Functional studies in *ex vivo* infected peritoneal macrophages indicate that the *Toxo1*-mediated restriction of parasite proliferation is associated with the coordinate death of both parasites and host macrophages. The parasite-induced macrophage cell death involved a caspase-1 dependent mechanism and exhibited pyroptosis-like features. The parasite-induced killing of infected macrophages could be blocked by the capase-1 inhibitor without restoration of parasite proliferation. Therefore, we concluded that if at work, the NLRP1a/caspase-1 pathway is not indispensable to restrict parasite proliferation in macrophages.

## Results

### Genetic dissection maps *Toxo1* to a <1 Mb region

**Figure 1:**
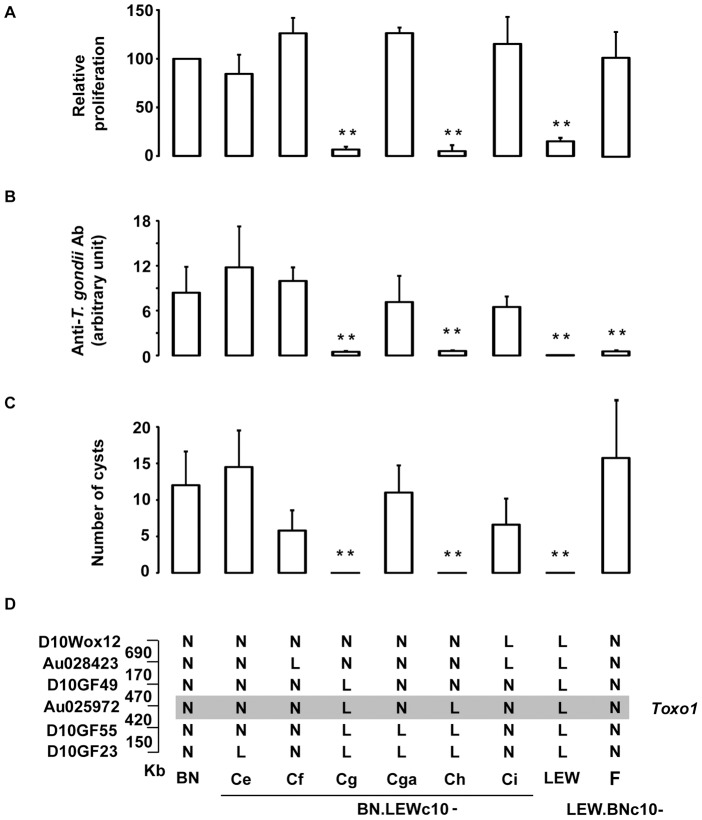
Genetic dissection narrows down *Toxo1* to 0.89 megabase. (**A**) Genetic dissection based on *in vitro* phenotypes. [^3^H] uracil incorporation into *T. gondii* RNA as a read-out of parasite proliferation within macrophages *in vitro*; results are normalized according to the values obtained in BN macrophages. Columns and bars show mean ± SD, n = 4 except LEW (n = 8) and BN.LEWc10-Cf (n = 3); _*_: *p*<0.05; _**_: *p*<0.01 as compared to BN). (**B–D**) Genetic dissection based on *in vivo* phenotypes. (**B**) Anti-*T. gondii* IgG response and cyst number were analyzed in BN, LEW and six congenic BN.LEWc10 lines. Anti-*T. gondii* IgG Ab response was analyzed by ELISA at day 30 post infection. (**C**) Number of brain cysts was determined by epifluorescence at day 60. (**C–D**) Columns and bars show mean ± s.e.m, n = 5 except for BN.LEWc10-Ce and Cga (n = 4); _*:_ *p*<0.05; _**:_ *p*<0.01 (as compared to BN). (**D**) Genotypes at the markers of the *Toxo1* locus in the used BN.LEWc10 congenic lines. The grey zone indicates the *Toxo1* locus narrowed down to 0.89 Mb (boundary markers: D10GF49 and D10GF55; physical distances are indicated between markers in kilobases).

We previously demonstrated that the *Toxo1* 7.6 Mb interval fully controls the outcome of *T. gondii* infection independently of the genetic background. The refractoriness to infection conferred by the LEW origin of *Toxo1* is characterized by the early elimination of the pathogen resulting in a barely detectable specific immune response and in the absence of brain cysts [10]. *In vitro*, this *Toxo1*-LEW mediated refractoriness is associated with the control of parasite proliferation within macrophages [10]. To refine the localisation of the gene(s) that control(s) these *in vivo* and *in vitro* phenotypes, we generated a unique panel of congenic sub-lines. Results from the genetic dissection are shown on Figure 1. The parasites were found able to proliferate within the macrophages from the congenic BN.LEWc10-Ce, -Cf, -Cga, -Ci and LEW.BNc10-F sub-lines but not within the macrophages from the congenic BN.LEWc10-Cg and -Ch sub-lines (Figure 1A). Thus within the 7.6 Mb of the *Toxo1* locus a 891 kb region controls the *in vitro* proliferation of parasites within macrophages. We further investigated refractoriness or susceptibility to *T. gondii* infection *in vivo* in rats from the seven congenic sub-lines used for these *in vitro* studies as well as in rats from the BN and LEW parental strains. The control of refractoriness to *T. gondii* defined by both the absence or low specific antibody response (Figure 1B) and the absence of cyst burden in the brain (Figure 1C), was directed by the same 891 kb region (Figure 1D). Thus, the interval located between the D10GF49 (57.26 Mb) and D10GF55 (58.15 Mb) microsatellite markers contains the gene or the set of genes that controls the toxoplasmosis outcome.

### Innate refractoriness against *T. gondii* is a common trait of several inbred rat strains

**Figure 2:**
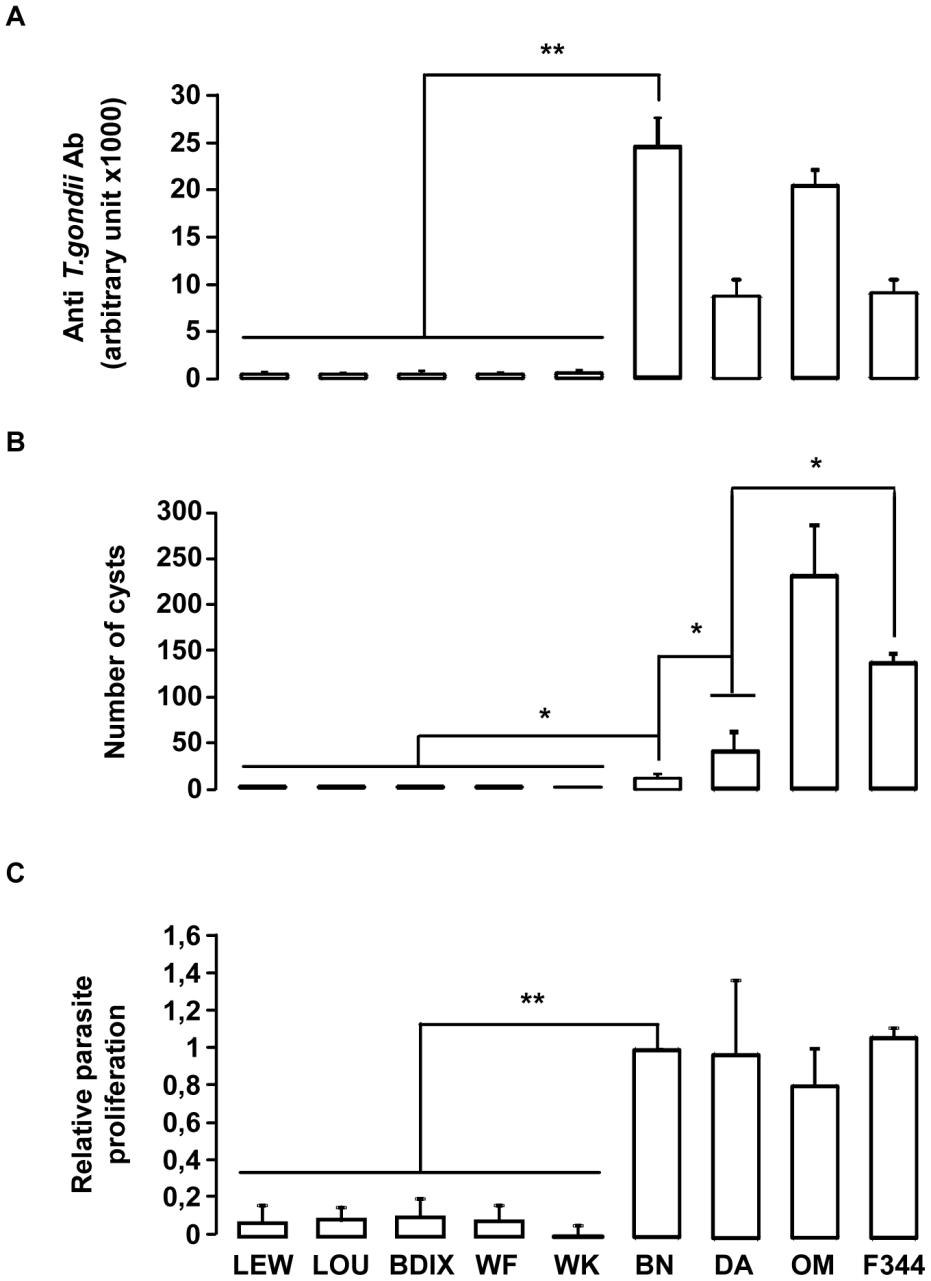
*In vivo* and *in vitro* phenotypes of the nine inbred strains following *T. gondii* infection. (**A–B**) *In vivo* phenotypes of the nine inbred strains. Anti-*T. gondii* IgG response and cyst number were analyzed in four rats from each inbred strain after oral infection of 20 cysts. (**A**) Anti-*T. gondii* IgG Ab were quantified by ELISA at day 30 post-infection. (**B**) Number of brain cysts was determined by epifluorescence and confirmed by real time PCR at day 60. Columns and bars show mean ± SD; _*_: *p*<0.05; _**_: *p*<0.01. (**C**) The intracellular growth of *T. gondii* in macrophages was determined by monitoring [^3^H] uracil incorporation. Results were normalized according to the values obtained for BN macrophages (100%). Columns and bars show mean ± SD of each group, n = 4 except LEW (n = 10) and BN (n = 10); _*_: *p*<0.05.

We hypothesized that genetic variation(s) underlying *Toxo1*-mediated innate refractoriness against *T. gondii* infection result(s) from an ancestral polymorphism instead of independent newly acquired mutations in the LEW strain and thus could be identified in various inbred rat strains. To challenge this hypothesis, we investigated toxoplasmosis outcome in seven other inbred rat strains (LOU, WF, WK, BDIX, OM, F344 and DA), in comparison to BN and LEW. Following infection, rats exhibited a dichotomous phenotype either developing high titers of anti-toxoplasma antibodies and cerebral cysts or remaining refractory to parasite infection (Figure 2). Three strains (OM, DA and F344) displayed, like the BN rat, phenotypes associated to chronic infection with high anti-*T. gondii* antibody responses (≥5000 u.a) and the detection of brain cysts. Of note, the number of brain cysts was significantly different among these rat strains (Figure 2B) reflecting other regulatory mechanism(s) for cyst formation. By contrast, the four other rat strains (LOU, BDIX, WK and WF) showed the refractory phenotype to *T. gondii* infection of the LEW strain neither developing specific antibodies nor cerebral cysts (Figure 2). As expected, parasite proliferation within peritoneal macrophages was observed only in the BN and the three other susceptible strains but neither in LEW nor in the four other refractory strains (Figure 2C). Thus, refractoriness against *T. gondii* infection is a common trait of several inbred rat strains.

### *Toxo1* controls resistance against *T. gondii* infection in the LOU rats

**Figure 3:**
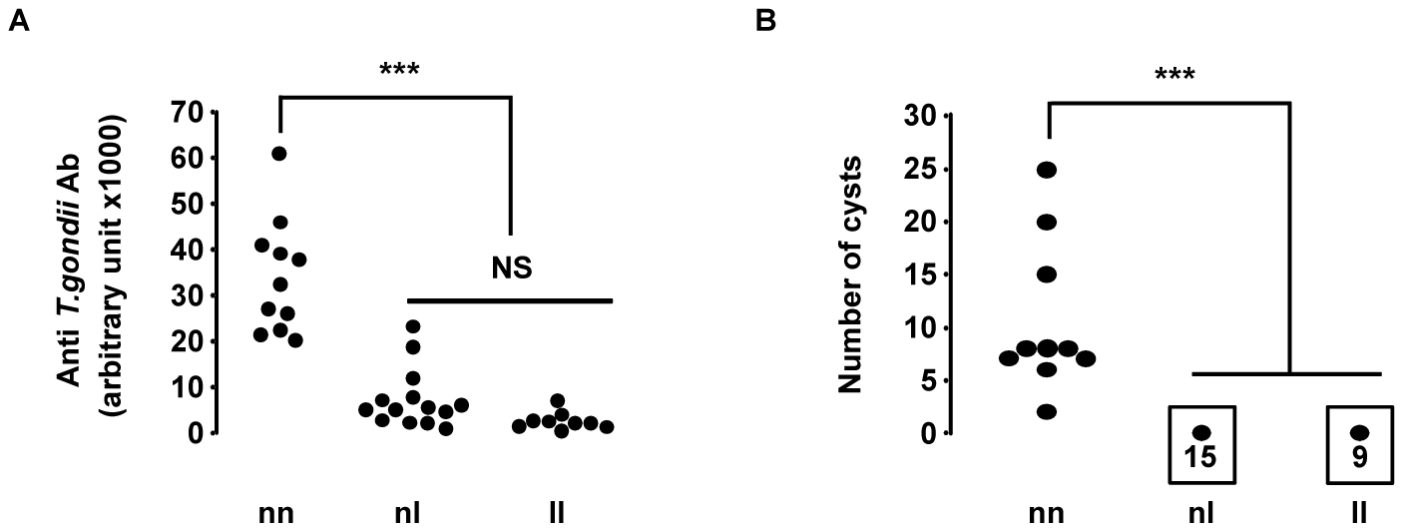
Susceptibility to *T. gondii* infection of (LOU X BN) F2 rats according to their genotype at the D10GF41 microsatellite marker. A total of 35 rats were studied, of which 9 were homozygous LEW (ll), 15 were heterozygous BN/LEW (nl), and 11 were homozygous BN (nn). (**A**) Anti-*T. gondii* Ab measured in arbitrary units by ELISA. Arbitrary units were established from a positive control. ll vs. nn and nl vs. nn: *P*<0.001. (**B**) Number of brain cysts determined by epifluorescence and confirmed by real time PCR. ll vs. nn and nl vs. nn, *P*<0.001.

To investigate the implication of the *Toxo1* locus in resistance against *T. gondii* infection, we used a targeted method of genotyping to stratify (LOU x BN) F2 rat according to their genotype at *Toxo1*. Thirty five F2 rats were genotyped at the polymorphic marker D10GF41, located at the peak of the *Toxo1* locus, and their anti-*T. gondii* Ab titers and brain cyst numbers were quantified following infection with *T. gondii* (Figure 3). All rats sharing the two LOU alleles (ll) showed no detectable or a weak anti-*T. gondii* Ab response (<10,000 a.u.). Conversely, all rats sharing the two BN alleles (nn) had high anti-*T. gondii* Ab titers (>20,000 a.u.). The apparent intermediate antibody response observed in the 15 heterozygous rats (nl) was not significantly different from that observed in the panel of rats sharing the LOU alleles (ll). In a similar way, brain cysts were observed in all the ten homozygous BN (nn) rats and in none of both the heterozygous BN/LOU (nl) and the nine homozygous LOU (ll). These results showed that *Toxo1* directs in a dominant manner the toxoplasmosis outcome in LOU rats after *T. gondii* infection.

### *Toxo1*-mediated refractoriness to toxoplasmosis is correlated with a high conservation of the LEW-genotype haplotype block

**Figure 4:**
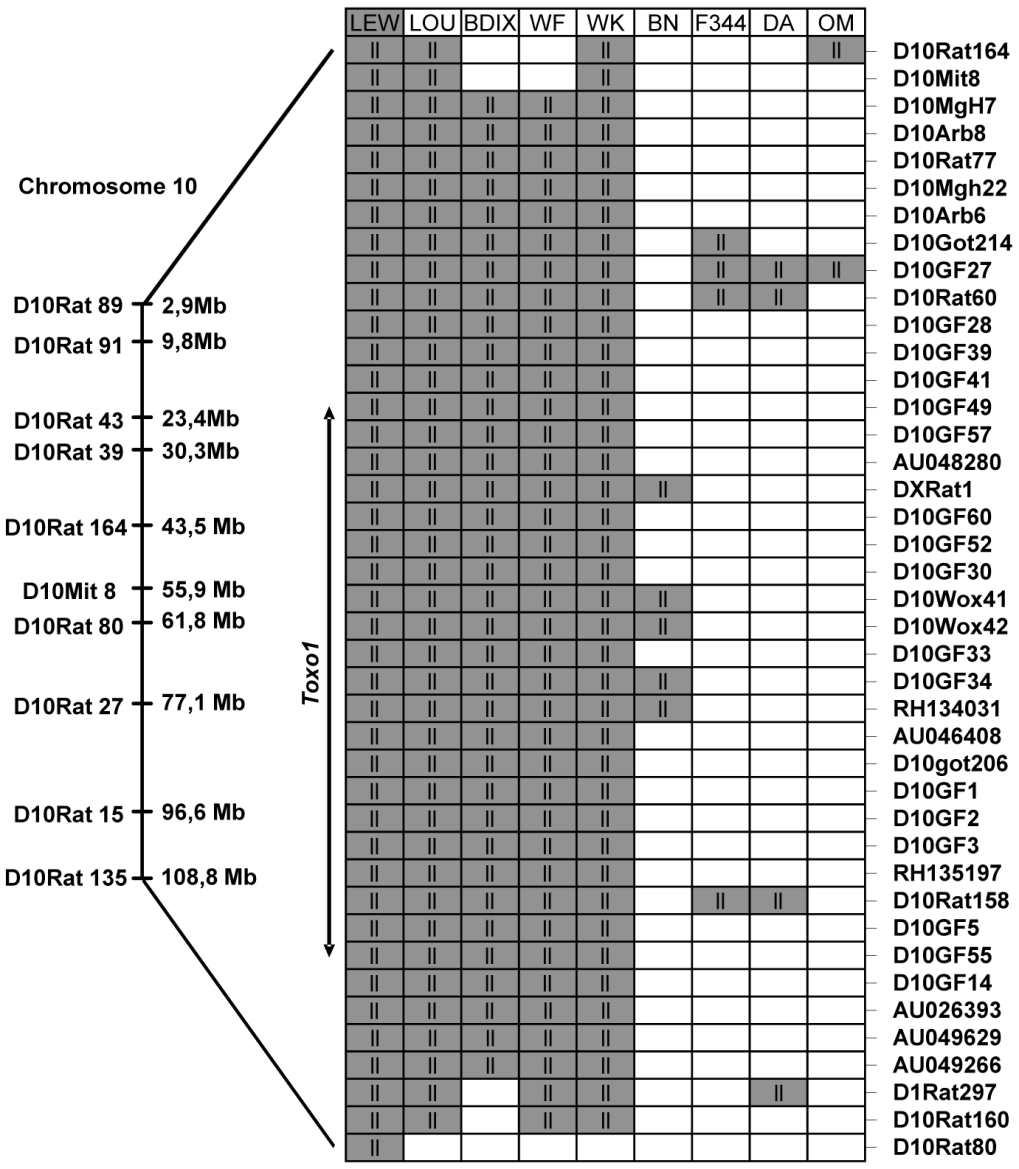
Haplotype analysis of the resistance locus to *T. gondii* infection, on chromosome 10. On the left, Markers with known locations in the genomic data for chromosome 10 of the Brown Norway rat (BN/SsNMcw; RGSC 3.4) are mapped to their approximate physical locations on the chromosome. Expansion indicates the region from D10Rat116 to D10Rat80 (7,6 Mb) corresponding to *Toxo1* locus previously identified [10] on chromosome 10. The right part represents the strain distribution patterns at several markers included between D10Arb7 and D1Rat297 on chromosome 10 (primers for all the markers are found in Figure S1). Genotypes are LEW-like (ll; gray) or different (white).

As *Toxo1* controls resistance against *T. gondii* infection in the LEW and LOU rats, and given the similarities between the response of LEW, LOU, BDIX, WK and WF, we next examined if *Toxo1*- allelic similarities are conserved among all those resistant animals. Considering that as a result of common ancestry, patterns of allelic similarities and differences among strains can be discerned for every variable locus [11], we conducted a haplotype study on the nine strains previously described. This analysis, based on allele size data for microsatellite markers, consisted in identifying among the different strains the chromosomal regions with LEW genotype. For this purpose, 41 polymorphic microsatellite markers on chromosome 10 were investigated on these nine strains (Table S1). The genotype of the nine strains for these markers showed the conservation of an haplotype block in the five resistant strains (LEW, LOU, WF, WK and BDIX) between D10Arb7 and D1Rat297 as compared to the four susceptible strains (BN, OM, F344 and DA) (Figure 4). This highly conserved haplotype block extends on 2.8 Mb between D10Arb7 and D1Rat297 and overlaps the entire 891 kb *Toxo1* locus (Figure 4). Thus, the data clearly suggested that conserved genetic variations within the *Toxo1* locus on chromosome 10 is a common cause of resistance against *T. gondii* infection in inbred rat strains.

### Sequencing of *Toxo1* revealed a densification of resistant-restricted conserved mutations in the region of *Nlrp1*

**Figure 5:**
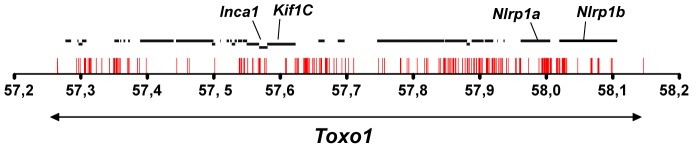
Allelic variations in *Toxo1* correlating with toxoplasmosis resistance. Sequencing of *Toxo1* (between 57.26 and 58.15 Mb) revealed 373 SNPs (red bars) conserved in coding- and non-coding sequences of all resistant strains and missing in susceptible strains. The diagram illustrates the distribution of these SNPs along the sequence of *Toxo1* (indicated by the double arrow in the lower part). In the upper part, the 29 genes are represented by black bold lines. The four genes named *Inca1*, *Kif1C*, *Nlrp1a* and *Nlrp1b* display missense mutations.

According to the genome database (www.ensembl.org, RGSC3.4 version), the *Toxo1*-891 kb interval contains 29 genes. None of these 29 genes could be retained as a candidate on the basis of a significant difference in their level of expression between macrophages from resistant *vs.* macrophages from susceptible congenic lines (Table S2, TextS1). Therefore, the entire *Toxo1* locus of the nine rat strains studied in the haplotype analysis was sequenced to identify resistance-correlated variations in coding- and non-coding sequences. A total of 373 SNPs and 21 insertions/deletions were found strictly conserved among the five resistant strains as compared to susceptible strains. The distribution of these mutations along the locus displays a gradient with a densification at the bottom of *Toxo1* (Figure 5). We identified 23 SNPs of which 16 are missense and one is a deletion in the coding sequences leading to the selection of four candidate genes: *Inca1* (1 SNP), *Kif1C* (1 ins/del), *Nlrp1a* (13 SNPs) and *Nlrp1b* (2 SNPs). Given that *Inca1* and *Nlrp1b* mRNAs are undetectable in peritoneal macrophages (Table S2) and according to the number of mutations in *Nlrp1a vs Kif1C* coding sequences, *Nlrp1a* appeared as the major candidate gene. It encodes the NOD-like receptor (NLR) NLRP1 that acts as an intracellular pattern recognition receptor (PRR) [12]. Both the mouse *Nlrp1b* and its ortholog rat *Nlrp1a* have been described as implicated in the control of a cell death process called pyroptosis that is induced by the lethal toxin (LT) from *Bacillus anthracis* [13].

### Early death of *T. gondii* within the parasitophorous vacuole is under the control of *Toxo1*

**Figure 6:**
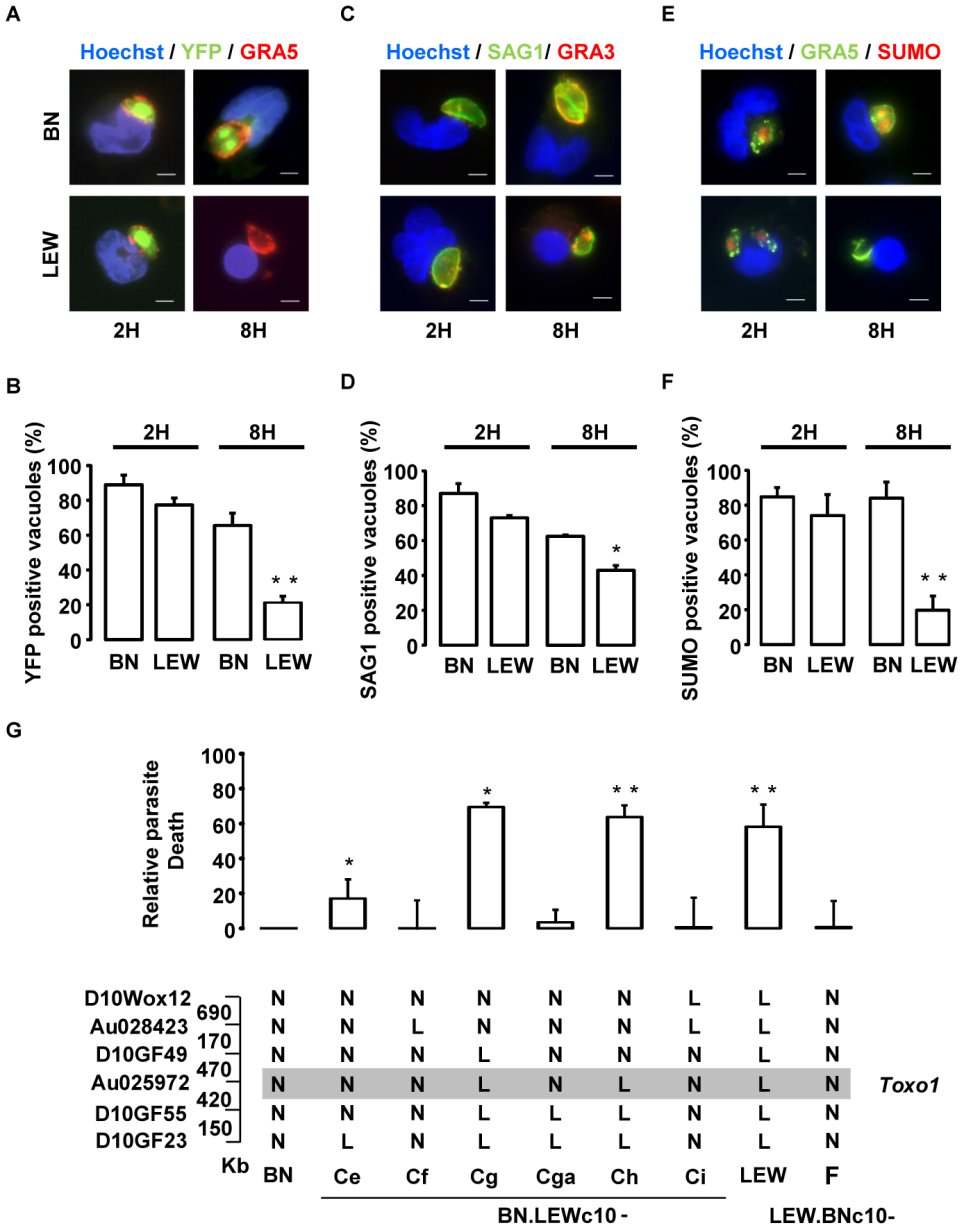
Intravacuolar Toxoplasma parasites are killed within resistant *Toxo1*-LEW peritoneal macrophages. (**A, B**) Macrophages settled on coverslips were infected with RH-YFP_2_ (green) parasites for 2 h or 8 h, fixed and stained with anti-GRA5 (red) and Hoechst 33258 (blue); **A**: representative IF images (Bars: 5 µm); **B**: Quantification of GRA5-positive vacuoles containing YFP-positive parasites (mean ± SD of three experiments; _**_: *p*<0.01). (**C, D**) Macrophages on coverslips were infected with RH parasites for 2 h or 8 h prior to fixation and stained with anti-SAG1 (green), anti-GRA3 (red) and Hoechst 33258 (blue). **C**: Representative IF images (Bars: 5 µm); **D**: Quantification of GRA3-positive vacuoles containing SAG1-labelled parasites (mean ± SD of two experiments; _*_: *p*<0.05). (**E, F**) Macrophages on coverslips were infected with RH parasites for 2 h or 8 h prior to fixation and stained with anti-GRA5 (green), anti-Tg small ubiquitin-like modifier (SUMO, red) and Hoechst 33258 (blue). **E**: representative IF images (Bars: 5 µm); **F**: Quantification of GRA5-positive vacuoles containing SUMO-positive parasites (mean ± SD of three experiments; _**_: *p*<0.01). (**G**) Rat macrophages from congenic lines were infected by the RH-YFP_2_ parasite strain. The YFP-positive infected cells were monitored by cytometry. Parasite death was evaluated through the ratio of YFP-positive infected cells between 6 h and 1 h post-infection. Results obtained for the LEW (n = 8) and congenic lines BN.LEWc10-Ce (n = 3), -Cf (n = 3), -Cg (n = 2), -Cga (n = 4), -Ch (n = 4), -Ci (n = 2), LEW.BNc10-F (n = 5) were normalized to the BN (n = 5) and represented by column showing means +/− SD; _*_: *p*<0.05; _**_: *p*<0.01. Genotypes at the markers of the *Toxo1* locus in the used BN.LEWc10 congenic lines are specified (N: homozygous for the BN genome; L: homozygous for the lew genome) and the grey zone indicates the *Toxo1* locus narrowed down to 0.89 Mb (boundary markers: D10GF49 and D10GF55; Physical distances are indicated between markers in kilobases).

Based on NLRP1 known function, we investigated the impact of the *Toxo1* locus on parasite and host cell fate after *ex vivo* infection of peritoneal macrophages. Following host-cell invasion, *T. gondii* replicates within a newly formed non-fusogenic compartment, the parasitophorous vacuole (PV). Using fluorescent parasites and vacuole staining with anti-GRA5 or -GRA3 antibodies which both stain the PV membrane, we compared the fate of intravacuolar parasites by immunofluorescence microscopy after allowing them to invade either non-permissive LEW or permissive BN naive peritoneal macrophages (Figure 6). The analyses were performed at 2 and 8 hours following invasion using transgenic-YFP_2_ fluorescent parasites. According to our previous work [10] we found similar rates of parasite invasion in both permissive (*Toxo1*-BN) (15%±5%) and non-permissive (*Toxo1*-LEW) (17%±6%) naive peritoneal macrophages. At 2 hours post-infection, most of intracellular YFP_2_-parasites were found within a compartment positive for the GRA5 PV membrane marker, in both LEW and BN macrophages (Figure 6A) indicating that parasites are able to enter efficiently into resistant macrophages. However, we observed a slight decrease in the percentage of YFP- and GRA5- positive vacuoles within LEW (77%±4) as compared to BN (89%±6) macrophages (Figure 6B). At 8 hours post-infection, while YFP_2_-parasites started to divide within GRA5 positive vacuoles of permissive BN macrophages (Figure 6A), a dramatic drop of YFP staining was observed in GRA5-positive vacuoles of LEW (21%±4) as compared to BN (66%±6) macrophages (Figure 6A and B). The loss of YFP emission in GRA5-positive vacuoles was likely to reflect the death of parasites [14] although parasite egress could not be excluded. Interestingly, the difference was not so marked when the parasite surface was stained using anti-SAG1 antibodies. Indeed, in BN macrophages, the percentage of vacuoles containing SAG1-positive parasites (62.5%±1) was not statistically different from the percentage of vacuoles containing YFP_2_-parasites (66%±6). In contrast, in LEW macrophages the percentage of vacuoles containing SAG1-positive parasites (43%±3) was two-fold the percentage of vacuoles containing YFP_2_-parasites (21%±4), indicating that at least 2/3 of vacuoles in LEW macrophages still contained parasites (Figure 6C and D). Therefore, the decrease of YFP staining could be attributed, at least in majority, to parasite death resulting from the macrophage microbicidal activity, rather than to parasite egress. The parasite death within the PV was further examined using staining of small ubiquitin-related modifier (SUMO) as a read-out of the transcriptional activity of live parasites [15]. As shown in Figure 6E and 6F, 2 and 8 hours after infection, a dramatic drop in the percentage of SUMO positive vacuoles was observed in LEW macrophages while no difference was found in BN macrophages, thus supporting the early death of parasites within LEW macrophages. We finally examined whether this phenotype was under the *Toxo1* control with our collection of sub-congenic rat strains. For this purpose, peritoneal macrophages from the four susceptible (BN.LEWc10-Ce, -Cf, -Cga, -Ci and LEW.BNc10-F) and the two refractory (BN.LEWc10-Cg and -Ch) sub-congenic lines were infected with *T. gondii*. In macrophages from the LEW and BN.LEWc10-Cg, -Ch lines in which *Toxo1* is from LEW origin, the inhibition of parasite proliferation correlates with the induction of intracellular parasite death (LEW: 58±13%; BN.LEWc10-Cg: 69±2%; -Ch: 64±7%). Conversely, in macrophages from the BN.LEWc10-Ce, -Cf, -Cga and -Ci lines in which *Toxo1* is from BN origin, the parasite proliferation was associated with a decrease of parasite death (BN.LEWc10-Ce: 17±11%; -Cf: 0±21%; -Cga: 3±7%; -Ci: 1±17%; LEW.BNc10-F: 0±16%) (Figure 6G). Altogether these results demonstrated that the *Toxo1* locus from LEW origin mediates the *T. gondii* killing by infected peritoneal macrophages.

### *T. gondii* triggers host macrophage death in a *Toxo1*-dependent way

**Figure 7:**
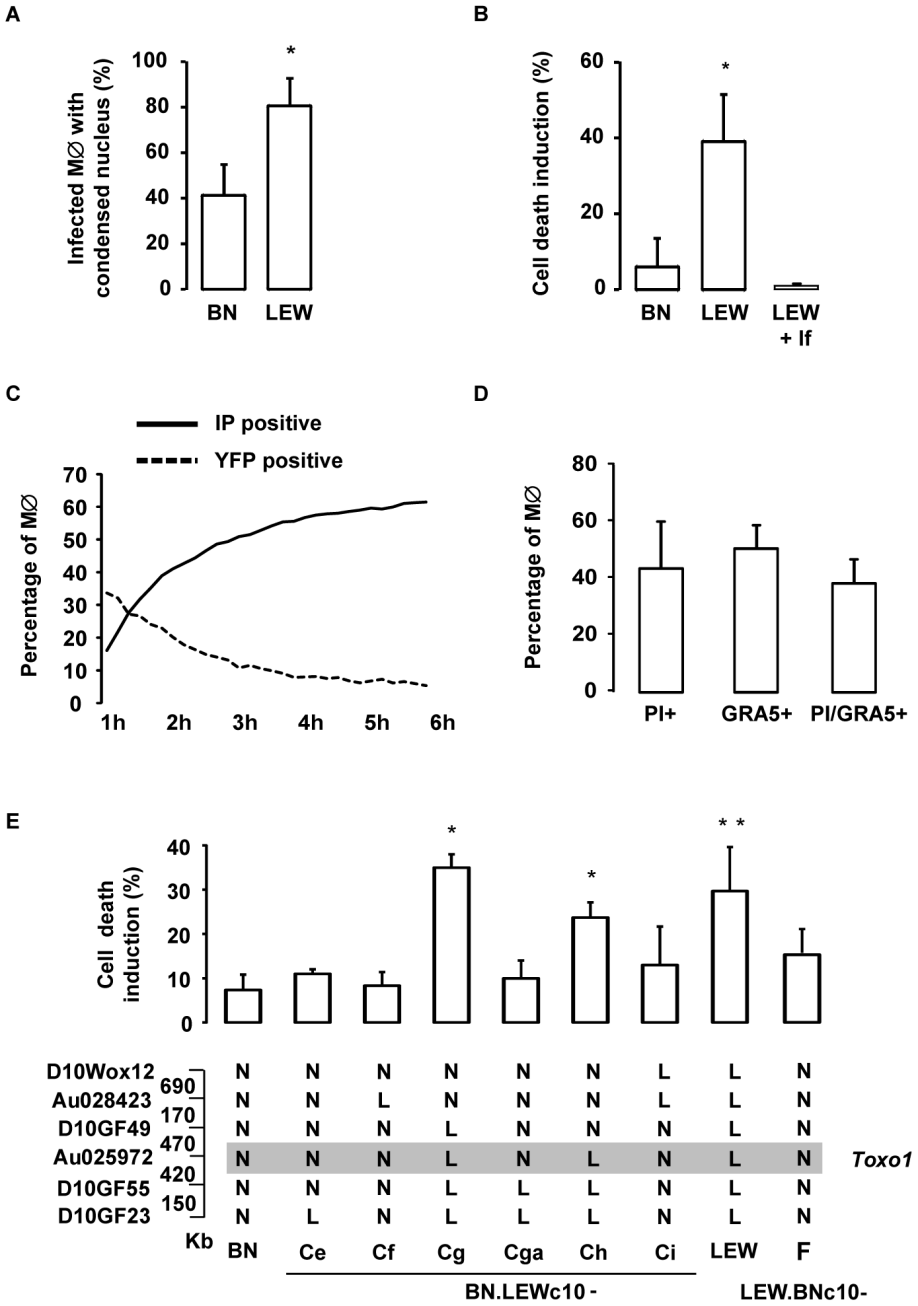
*T. gondii* killing is associated with the rapid death of infected *Toxo1*-LEW macrophages. (**A**) Percentage of infected BN and LEW macrophages showing a condensed nucleus, 8 h after infection. Columns represent mean +/− SD of three different experiments. (**B**) Cell death was monitored by propidium iodide (PI) uptake. Percentage of induced-cell death on BN and LEW peritoneal macrophage, 6 h after either infection (n = 4 for BN and LEW) or incubation with lysed fibroblasts (lf) (n = 2). Results represent differences between infected and uninfected cells. Columns represent mean +/− SD. _*_: *p*<0.05. (**C**) Death kinetics of both intracellular RH-YFP parasites and host macrophages. Parasite death was assessed by the percentage of YFP positive cells (dotted line), and host macrophage death by the percentage of PI positive cells (solid line). (**D**) Cell death induction is restricted to infected macrophages. LEW macrophages were infected with RH parasites for 6 h prior to fixation and stained with anti-GRA5 antibody, PI and Hoechst 33258. Columns represent mean +/− SD of two different experiments. (**E**) The percentage of cell death induction, in peritoneal macrophages, from BN (n = 7), LEW (n = 8) and congenic lines BN.LEWc10-Ce (n = 3), -Cf (n = 3), -Cg (n = 4), -Cga (n = 4), -Ch (n = 4), -Ci (n = 3), LEW.BNc10-F (n = 5) was monitored by PI uptake. Columns and bars show means +/− SD; _*_: *p*<0.05; _**_: *p*<0.01. Genotypes at the markers of the *Toxo1* locus in the used BN.LEWc10 congenic lines are specified (N: Homozygous for the BN genome; L: Homozygous for the LEW genome) and the grey zone indicates the *Toxo1* locus narrowed down to 0.89 Mb (boundary markers: D10GF49 and D10GF55; Physical distances are indicated between markers in kilobases).

We next examined the fate of infected host cells in a comparative way depending on the *Toxo1* genotype. At 8 hours post-infection, we observed that most of LEW macrophages infected with YFP-negative vacuoles presented a condensed nucleus (Figures 6A, 6C, 6E). Indeed, when this phenomenon was quantified we found that 65% of LEW macrophages with YFP-negative vacuoles, but only 14% of BN susceptible macrophages, showed a condensed nucleus (Figure 7A). This observation indicating a parasite-induced killing of LEW macrophages was further investigated using propidium iodide (PI) uptake. At 6 hours post-infection, about 40% of LEW macrophages (39%±12) had lost membrane integrity as compared to less than 10% of BN macrophages (6%±8) (Figure 7B, p<0.05). The lack of PI uptake under incubation with lysed fibroblasts ruled out the possible triggering by unrelated pathogen-associated molecular pattern (PAMP) (Figure 7B). Tracking of both PI uptake and YFP loss in kinetic experiments indicated that the parasite-induced cell death of LEW macrophages is concomitant with the death of intracellular parasites. Indeed, the increase of PI uptake by macrophages and the loss of YFP by intracellular parasites started from 1 hour and increased steadily until 6 hours after infection (Figure 7C). Further quantification of PI and GRA5 PV marker positive LEW macrophages demonstrated that 88% of dying cells were parasite-invaded cells (Figure 7D). Finally, the implication of *Toxo1* in the host cell death phenotype was validated using our panel of sub-congenic animals. Specific induction of host cell death after infection of non-permissive peritoneal macrophages was found in all resistant congenic rats (LEW: 30±10%; BN.LEWc10-Cg: 35±3%; -Ch: 24±3%). By contrast, peritoneal macrophages from all animals permissive to *T. gondii* proliferation failed to initiate such a cell death process after infection (BN.LEWc10-Ce: 11±1%; -Cf: 8±3%; -Cga: 10±4%; -Ci: 13±7% and LEW.BNc10-F: 17±5%) (Figure 7E). Altogether, these data demonstrated that *T. gondii* invasion of resistant macrophages is rapidly followed by the combined deaths of intracellular parasites and infected host macrophages using a mechanism under the control of *Toxo1* locus.

### The *Toxo1*-mediated death of *T. gondii*-infected macrophages is neither apoptosis nor autophagy

**Figure 8:**
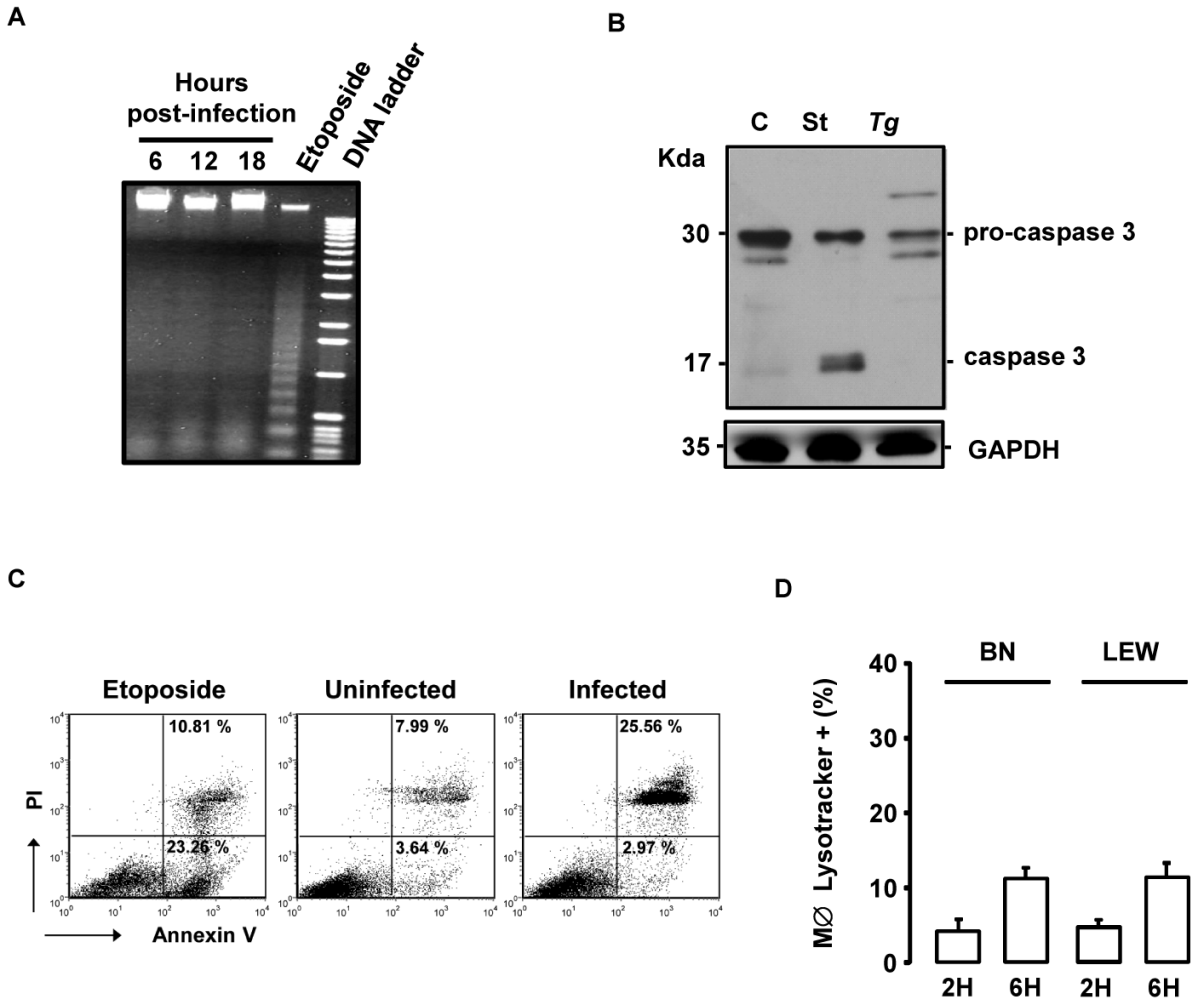
The *T. gondii*-induced death of infected LEW macrophages is neither apoptosis nor autophagy. (**A**) *T. gondii*-induced death of LEW macrophages does not show DNA fragmentation. LEW macrophages were either infected with RH-YFP_2_ parasites for 6 h (lane 1), 12 h (lane 2) or 18 h (lane 3) or treated with 50 µM etoposide for 18 h (lane 4). The 1 Kb DNA ladder as marker is shown in lane 5. (**B**) *T. gondii* does not trigger the processing of caspase-3 in LEW peritoneal macrophages after infection. Cleavage of pro-caspase 3 was determined by western blotting of lysates from untreated LEW macrophages (C) or following staurosporine treatment (St) or *T. gondii* infection (Tg). (C) Representative flow cytometry dot plots of rat peritoneal macrophages stained with propidium iodide and annexin V antibody. LEW macrophages were either uninfected (medium) or infected 4 h with *T. gondii* (right) and compared to rat macrophages treated 4 h with etoposide (left). (**D**) BN and LEW macrophages settled on coverslips, infected with RH-YFP_2_ parasites and stained with LysoTracker Red DND-99 2 h and 6 h post-infection. Each column represents mean +/− SD of three different experiments.

The rapid loss of membrane integrity of parasite-invaded LEW macrophages (Figure 7C) suggested that the *T. gondii*-induced cell death was not apoptotic. Accordingly, DNA from dying cells did not show the laddering resulting from chromatin fragmentation, observed in classical apoptotic cell death and pyroptosis (Figure 8A). Moreover, caspase-3 activation, which plays a central role in the executive phase of apoptosis, was not observed in infected LEW macrophages (Figure 8B). Additionally, when infected macrophages were incubated with FITC-labelled annexin V, phosphatidylserine exposure on the plasma membrane was not observed prior to the loss of membrane integrity as assessed by PI staining (Figure 8C). Finally, no difference in the number of acidic vacuoles was observed after lysotracker coloration between permissive BN and non-permissive infected LEW macrophages indicating that the death of infected LEW macrophages was not due to autophagy (Figure 8D).

### The *Toxo1*-mediated death of *T. gondii*-infected macrophages is associated to ROS production and caspase-1 activation

**Figure 9:**
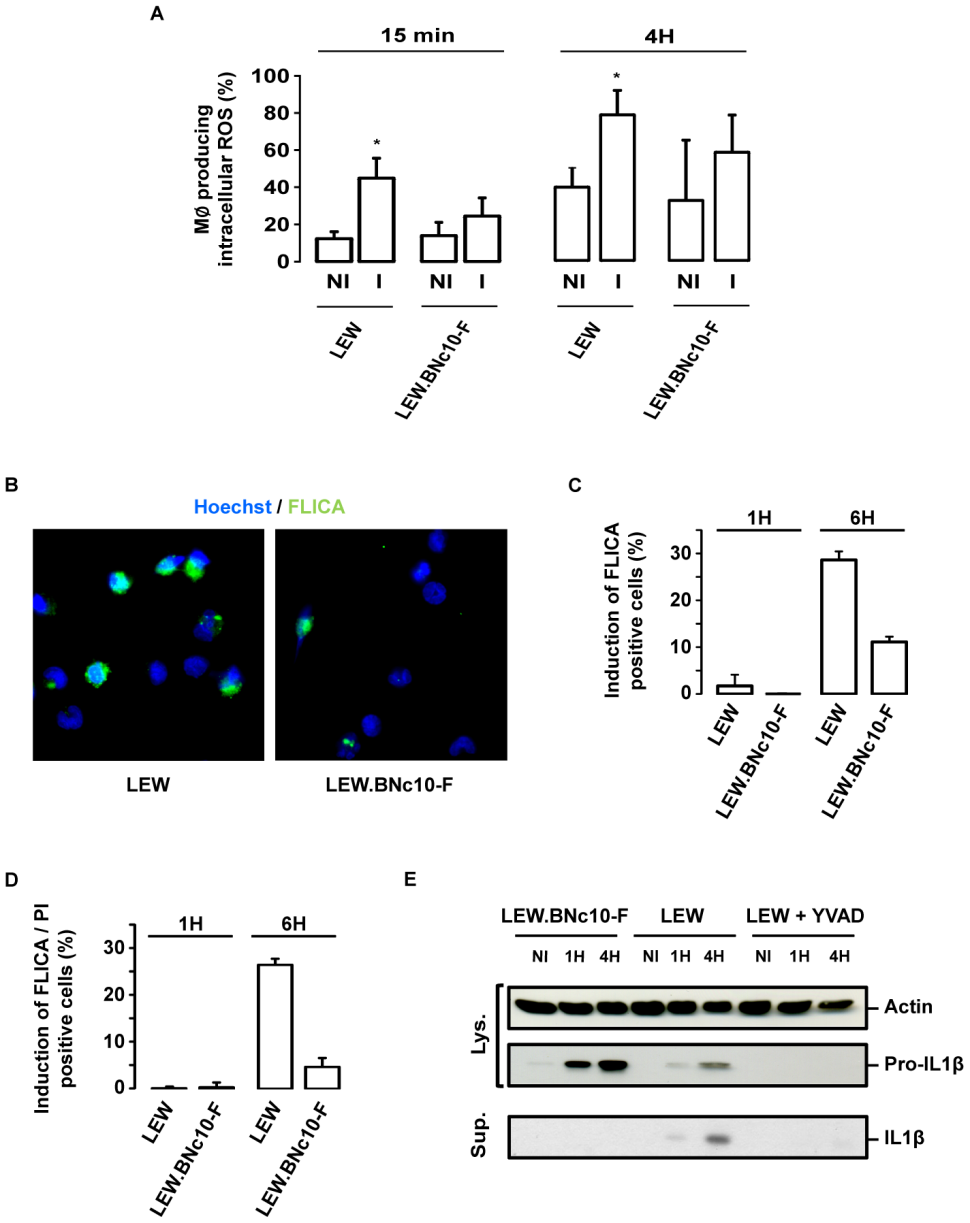
The *Toxo1*-mediated death of *T. gondii*-infected macrophages is associated to ROS production and caspase-1 activation. (**A**) LEW and LEW.BNc10-F were infected 15 min or 4 h with *T. gondii*, then dihydro-rhodamine was added, and 15 min later the percentage of cells producing ROS was determined by flow cytometry. Histograms represent the percentage of cells producing ROS in uninfected and infected macrophages. Columns and bars show mean ± SD of three independent experiments; _*_, *p*<0.05. (**B**) Representative IF images of LEW and LEW.BNc10-F macrophages infected 4 h with RH parasites and labelled with FLICA assay (green) prior to fixation and Hoescht staining (blue). (**C, D**) LEW and LEW.BNc10-F macrophages were infected for 1 h and 4 h with RH parasites and then labelled with FLICA assay (green) and PI (red) prior to fixation and Hoescht staining (blue). (**C**) Histograms represent analysis of FLICA positive cells from two independent experiments. Data are means +/− SD. (**D**) Histograms represent analysis of FLICA and PI positive cells from two independent experiments. Data are means +/− SD. (**E**) *T. gondii* triggers caspase-1 dependent processing of IL-1β in resistant LEW but not in permissive Lew.BNc10-F macrophages. IL-1β precursor and mature IL-1β were analyzed by western blotting of cell lysates (Lys.) and culture supernatants (Sup.) from LEW and Lew.BNc10-F macrophages uninfected (NI) or infected for 1 h or 4 h with or without 50 µM of capase-1 inhibitor YVAD (Calbiochem). Immunoblot was normalized using equivalent number of cells for each condition and validated with anti-actin immunoblot.

Given the described role of NLRP1/Caspase-1 inflammasome pathway in the host response to pathogens [16], [17], we hypothesized that the *T. gondii*-induced cell death of infected resistant LEW macrophages could be associated with both ROS production and caspase-1 activation. The production of intracellular ROS by macrophages was monitored at two time points (15 min and 4 hours) by the dihydro-rhodamine 123. While *T. gondii* infection did not induce significant ROS production within permissive LEW.BNc10-F macrophages, a marked increase was recorded in infected resistant LEW macrophages (Figure 9A). We next examined caspase-1 activation within infected macrophages by using the fluorogenic activated caspase-1 specific staining (FLICA). At 4 hours post-infection, the percentage of parasite-induced FLICA positive cells was significantly higher in resistant LEW macrophages (29%±2) than in permissive LEW.BNc10-F macrophages (11%±1) (Figure 9B and C). The caspase-1 induction in infected LEW macrophages correlated with the increase of PI-positive cells indicating that caspase-1 was involved in the cell death induction process (Figure 9D). Consistent with these results, the processing of caspase-1 substrate IL-1β that could be prevented by the YVAD caspase-1 inhibitor was detected at 1 h and more evidently at 4 h post-infection in the culture supernatant of infected LEW macrophages and not in that of permissive LEW.BNc10-F macrophages (Figure 9E). The observed difference in the secretion of mature IL-1β was not due to a lack of pro-IL-1β expression since it was found to be induced in response to *T. gondii* infection within both resistant LEW and permissive LEW.BNc10-F macrophages (Figure 9E). By contrast, pro-IL-1β was not detectable in the cell lysate from LEW macrophages treated with YVAD (Figure 9E) indicating that the inhibition of caspase-1 activity resulted in the down-regulation of pro-IL-1β protein expression. Altogether, these results demonstrated that both ROS production and caspase-1/IL1β pathway are involved in the *Toxo1*-mediated cell death induction of resistant LEW macrophages.

### The caspase-1 pathway controls the cell death induction of both host macrophages and intracellular parasites but not parasite proliferation

**Figure 10:**
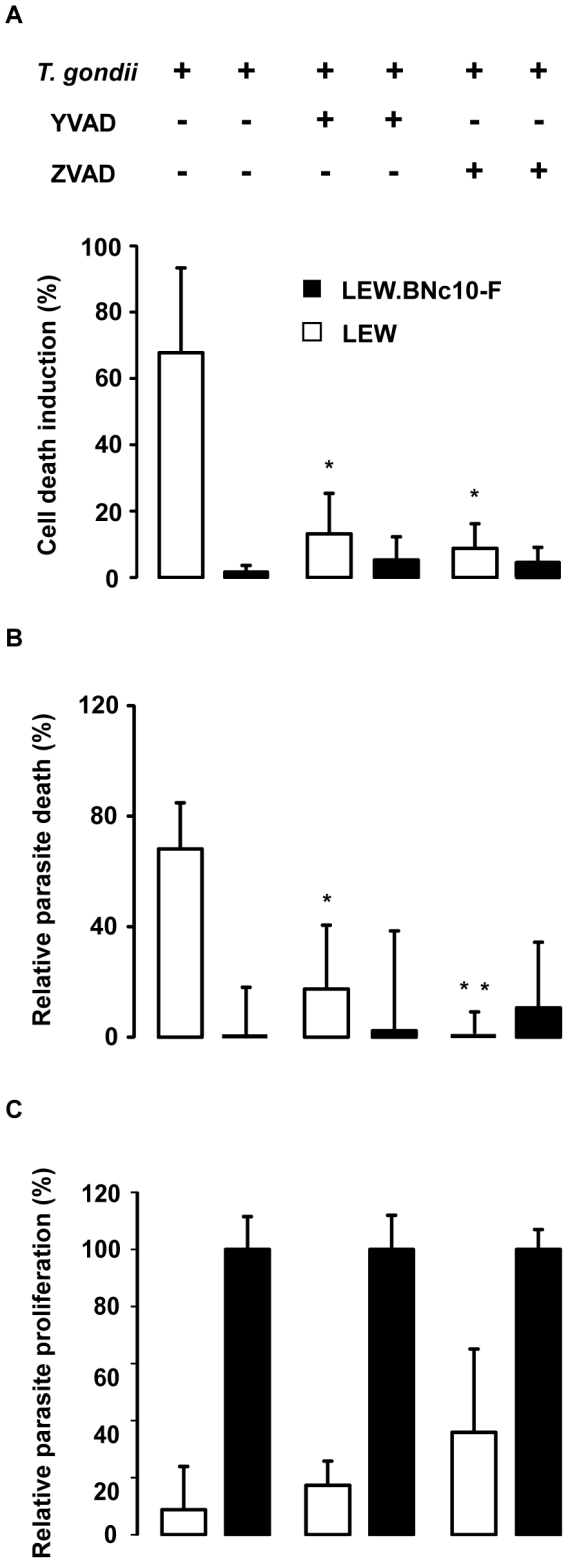
The caspase-1 pathway controls the cell death induction of both host macrophages and intracellular parasite but not parasite proliferation. (**A, B, C**) Caspase-1 inhibitor YVAD and pan-caspase inhibitor Z-VAD (Calbiochem) were applied at respectively 50 µM and 100 µM to LEW and LEW.BNc10-F peritoneal macrophages infected 6 h with RH-YFP parasites. (**A**) Cell death was monitored by PI uptake. Histograms represent the percentage of PI positive dying cells (results indicate the difference between infected and uninfected cells). Data are means +/− SD from three independent experiments; _*_: *p*<0.05. (**B**) The YFP-positive infected macrophages were monitored by fluorescent microscopy. Parasite viability was evaluated through the percentage of YFP-positive infected cells 6 h post-infection. Results were normalized according to the percentage of YFP-positive infected cells obtained in untreated LEW.BNc10-F macrophages. Columns represent mean +/− SD of three different experiments; _*_: *p*<0.05; _**_: *p*<0.01. (**C**) Intracellular growth of *T. gondii* was measured by monitoring [^3^H] uracil incorporation into *T. gondii*. For each treatment, results were normalized according to the values obtained in LEW.BNc10-F macrophages. Columns represent mean +/− SD of three independent experiments; _*_: *p*<0.05; _**_: *p*<0.01.

The role of caspase-1 in the resistance of LEW macrophages was further investigated using pharmacological inhibitors. Both caspase-1 and pan-caspase inhibitors were able to protect the resistant LEW macrophages from the parasite-induced cell death (Figure 10A). Consistent with our above observations that both parasites and host macrophages killing processes are connected, the two inhibitors also prevented the death of intracellular parasites (Figure 10B). By contrast, these inhibitors failed to restore significant parasite proliferation in non-permissive macrophages (Figure 10C). Altogether these experiments revealed that the resistance of macrophages bearing *Toxo1*-LEW alleles relies on two pathways, the first one controlling parasite proliferation and the second one controlling the death of both intracellular parasites and host macrophages, via caspase-1 dependent inflammasome activation.

## Discussion

Forward genetics has proved to be a powerful tool to characterize novel biological pathways implicated in host resistance to infection [18], [19]. In rats, the *Toxo1* locus located on chromosome 10 [10] controls the outcome of toxoplasmosis by a still poorly defined mechanism. In the present work, genetic dissection of *Toxo1* with a panel of new congenic sub-lines together with haplotype mapping led us to identify a 891 kb interval of rat chromosome 10 that is highly conserved amongst resistant rat strains and that controls both *T. gondii* infection outcome *in vivo* and macrophage responses to infection *in vitro*. We further demonstrated that the *Toxo1*-mediated refractoriness of macrophages to *T. gondii* infection is associated with a caspase-1-dependent rapid *T. gondii*-induced death of both intracellular parasites and host macrophages.

The *Toxo1*-dependent death of infected macrophages displayed the chromatin condensation hallmark of apoptosis but neither caspase-3 activation nor DNA fragmentation were observed. In contrast to apoptosis which is usually a slow process characterized by membrane blebbing, the *Toxo1*-associated cell death was characterized by a rapid loss of plasma membrane integrity that occurs simultaneously with surface exposure of phosphatidylserine.

A non-apoptotic pathway triggered in *T. gondii*-infected macrophages has been described in mice [20]. It is mediated by IFN-γ-inducible immunity-related GTPases that trigger vacuole membrane disruption and the death of parasites, followed by the necrotic-like death of infected cells. While phenotypically, the IRG-dependent mouse *T. gondii*/macrophage deaths parallel the *Toxo1*-mediated *T. gondii*/macrophage deaths, major differences between the two *in vitro* models exist, providing strong evidences that mechanistically they are not identical. The mouse IRG-controlled mechanism requires IFN-γ stimulation and occurs in both fibroblasts and macrophages. In contrast, the *Toxo1* effect in rats is strictly confined to hematopoeitic cells [9], [10] and does not depend on IFN-γ stimulation of macrophages *in vitro*. Moreover, the mouse IRG-controlled resistant system is genotypically restricted to non-virulent genotype II parasites, while in rats, both type I RH and type II PRU strains triggered the LEW *T. gondii*/macrophage deaths (Figure S1). In line with this, the effector of the LEW rat resistance is not the kinase parasite effector ROP 18 (data not shown) which, by phosphorylating IRGs, disrupts their association with the parasite-containing vacuole and thereby protects the parasite against elimination [21], [22]. Altogether, the rat *Toxo1* locus-mediated resistance to toxoplasmosis does not operate via the IRG-dependent system.

Sequencing of the *Toxo1* region in all five resistant rat strains (LEW, LOU, BDIX, WK and WF) and four susceptible strains (BN, OM, DA and F344) revealed a gradient in the conserved distribution of resistant-restricted mutations that peaks at the bottom of *Toxo1* and particularly in the *Nlrp1a* coding sequence (23 SNPs). This highly conserved region has been previously demonstrated to be associated with the resistance of rats to the lethal Toxin (LT) of *Bacillus anthracis* [23]. Interestingly, while rat bearing the divergent *Toxo1* alleles were susceptible to LT-mediated death, the highly conserved *Toxo1*-LEW alleles correlated with the total resistance of rats [23]. Moreover, similarly to what we found for the *Toxo1* control of *T. gondii* infectivity, there was a perfect correlation between the *in vivo* phenotype (sensitivity *vs* resistance to LT) and the *in vitro* phenotype of macrophages, suggesting that the same gene or set of genes might be at work in the control of these two pathogens. In mice and rats, further genetic mapping associated genetic variants of both *mNlrp1b* and one of its rat ortholog *rNlrp1a* to macrophage sensitivity or resistance to LT by a mechanism dependent on a cell death process called pyroptosis, which is also triggered upon infection with intracellular pathogens such as *Salmonella*, *Shigella* or *Listeria* [24]–[26]. NLRP1 is part of the NLR family, which is known to act as an intracellular sensor for cytoplasmic danger signals [12]. After activation, the NLR form a multimeric protein complex called the inflammasome that provides a scaffold for the activation of caspase-1 and target death substrates by a still poorly understood mechanism [12]. The *Toxo1* controlled-cell death induction that is triggered in peritoneal macrophages following *T. gondii* invasion, appeared to be also caspase-1 dependent and associated to IL-1β secretion and ROS production. Together, with the nuclear condensation, it thus features the hallmarks of the pyroptosis programmed-cell death which is uniquely dependent on caspase-1 and inherently proinflammatory. However, while pyroptosis is typically associated to DNA fragmentation [16], this later event was not observed in the *T. gondii*-induced cell death possibly due to a yet unexplained undetectable PARP (Poly ADP-Ribose Polymerase) expression in rat peritoneal macrophages (Figure S3, Text S1). Together these observations combined with the genetics studies tend to support that the NOD-like receptor NLRP1a/Caspase-1 pathway is the best candidate to mediate the *Toxo1*-dependent parasite-induced cell death.

Despite these remarkable genetic and biochemical similarities, LT- and *T. gondii*-induced phenotypes display striking major differences. First, while *Toxo1*-conserved LEW alleles are associated to the cell death induction of macrophages upon *T. gondii* infection, same allelic variants protect the macrophages from LT-mediated cell death [23]. Secondly, while the pharmacological inhibition of host cell death also prevents the concomitant death of parasites, it failed to restore the permissiveness of macrophages to parasite proliferation. Thus, although caspase-1 is induced following *T. gondii* infection, our data do not argue for its essential role in the macrophage control of parasite proliferation *in vitro*. They rather suggest that, like it is emerging in the case of intracellular bacteria, the *Toxo1*-directed resistance of macrophages to *T. gondii* proliferation may rather be a complex trait resulting from the combined activation of several pathways. For instance, the Naip5/Nlrc4-controlled restriction of *Legionella pneumophila* growth within non permissive murine macrophages is likely to involve both canonical pyroptosis and a yet undefined caspase-1 independent Naip5 pathway [27]. Very interestingly, in mouse macrophages infected by *Shigella flexneri* which display NLRC4- and NLRP3-dependent activation of caspase-1, IL-1β/IL-18 processing and cell death [17], [26], it has been demonstrated that inflammasome might negatively regulate pathogen-induced autophagy [26]. Given that other NLRs may affect autophagy [17], inflammasome inhibition of autophagy might be a generalized mechanism. Moreover, Harris J. and al. demonstrated that autophagy controls IL1-β secretion by targeting pro-IL1-β for degradation [28]. In line with this, the lack of detectable pro-IL1-β in resistant LEW macrophages treated with caspase-1 inhibitor, suggested that inactivation of caspase-1 results in the down-regulation of pro-IL1-β protein expression. It is therefore possible that in our model, the inhibition of caspase-1 could block the negative regulation of inflammasome promoting thus the effect of other pathway(s) capable to restrict parasite proliferation without pyroptosis. Altogether, our work provide evidence that natural resistance of rat macrophages to *T. gondii* is a complex trait relying on a mechanism involving the combined activation of at least two pathways: (i) the classical NLRP1a/Caspase-1 pathway, mediating host and parasite cell death and (ii) a yet undefined pathway that would directly control parasite proliferation within the parasitophorous vacuole. The parasite effector(s) eliciting these pathways and their possible interconnections remain to be investigated.

*Toxoplasma* infection is naturally acquired by the oral route. Following transcytosis across the intestinal barrier [29], the tachyzoite stage encounters leukocytes in which it replicates to further disseminate into the organism using the migratory properties of infected macrophages and dendritic cells [30]. In rats bearing the LEW-type *Toxo1* locus, the refractoriness to toxoplasmosis is evidenced by the absence of both local parasite burden and specific antibody response [9]. Thus resistance likely results from the rapid clearance of the parasite following a vigorous killing response at the site of infection. Our data led us to propose a model where the early death of infected macrophages impairs further dissemination of the parasite, hence constituting an efficient barrier against successful infection. How the *Toxo1* locus controls dendritic cell and monocyte responses after infection remains to be characterized.

In conclusion, this work highlighted several novel aspects of the host-parasite gene interaction. We unambiguously mapped the *Toxo1* locus to an 891 kb region which directs the outcome of toxoplasmosis in the rat. This locus controls parasite infectivity *in vivo* and is critically associated to macrophage-dependent restriction of parasite intracellular growth and cell death induction of both intracellular parasites and infected macrophages *in vitro*. It is included within a haplotype block remarkably subjected to strong selection pressure for resistant strains, in contrast to susceptible strains (Figure S2, Text S1). The robustness of *Toxo1*-mediated resistance in controlling parasite infectivity indicates that this resistance might have been conserved among naturally resistant species [11]. Hence, Transmission Disequilibrium Test studies revealed that *hNlrp1*, the human ortholog of rat *rNlrp1a*, has alleles associated with the susceptibility to human congenital toxopolasmosis [6]. In the same way, recent work demonstrated that, in mice, NLRP1 is an innate immune sensor for Toxoplasma infection inducing a host-protective innate immune response to the parasite [31]. Altogether, these data identified a genetically-controlled major pathway of innate immunity to toxoplasmosis allowing predicting resistance of individuals. The results open the way to further investigations towards the gene(s) and the mechanisms at work, and could be applied to human toxoplasmosis in regards to the conserved synteny of *Toxo1* region between rat and human.

## Materials and Methods

### Ethics statement

Breeding and experimental procedures were carried out in accordance with national and international laws for laboratory animal welfare and experimentation (EEC Council Directive 2010/63/EU, September 2010). Experiments were performed under the supervision of M–F. C–D. (agreement 38 10 38) in the Plateforme de Haute Technologie Animale (PHTA) animal care facility (agreement n° A 38 516 10006 delivered by the Direction Départementale de la Protection des Populations) and were approved by the ethics committee of the PHTA (permits n° Toxo-PC-1 and n° Toxo-PC-2).

### Rats and infection

Production and genotype analyses of congenic lines were as described previously [10]. The congenic lineages were maintained by regular brother-sister mating. LEW/OrlRj (LEW), BN/OrlRj (BN) male rats were obtained from Janvier Laboratory (Le Genest-Saint-Isle, France). WF/N (WF), WKY/NHsd (WK), BDIX/Han (BDIX), F344/Nhsd (F344), Lou/CNimrOlaHsd (LOU) and DA/OlaHsd (DA) male rats were obtained from Harlan Laboratory (Gannat, France). OM/Han (OM) rats were kindly supplied by the Hannover Medical School (Germany). F2 (LOU×BN) progenies were produced in our animal facilities under specific pathogen-free conditions. Cysts from the recombinant *T. gondii* Prugniaud strain were used to test *in vivo* the susceptibility to toxoplasma infection. Two-month-old Swiss mice (Janvier laboratory) were infected orally with 10 Prugniaud cysts. Their brains were collected 3 months later and ground in a Potter. Cysts were counted in a Thoma's cell and diluted in PBS. Rats were infected orally with 20 cysts. One month later, blood was collected from the retro-orbital sinus for detection of anti-toxoplasma Ab response by ELISA. Rats were euthanized 2 months after infection and brains were collected to determine number of cysts.

### Parasites

Tachyzoites of Prugniaud type II strain, and RH, RH-YFP_2_ (kindly provided by B. Striepen, Athens) and RH-mcherry (kindly provided by A. Bougdour, Grenoble) type I *T. gondii* strains were maintained under standard procedures, by serial passage onto human foreskin fibroblast monolayers (HFFs) in D10 medium (DMEM supplemented with 10% heat-inactivated fetal bovine serum, 1 mM glutamine, 500 units.ml^−1^ penicillin and 50 µg.ml^−1^ streptomycin) at 37°C in a humidified atmosphere containing 5% CO_2_. The parasites were collected just before the experiment, centrifuged at 500× *g* for 7 min, suspended in serum-free medium (SFM, GIBCO) supplemented with 500 units.ml^−1^ penicillin and 50 µg.ml^−1^ streptomycin, and counted.

### Peritoneal macrophages and infection

Rat resident peritoneal cells were obtained by injection of sterile PBS into the peritoneal cavity. Collected cells were centrifuged and resuspended in Serum Free Medium (SFM) (Life Technologies, Inc) and counted. Macrophages were obtained by adhering cells for 1 h at 37°C and 5% CO_2_. After 1 h, non-adherent cells were removed by gentle washing with SFM and parasites were added to macrophages settled on coverslips at a ratio of 3∶1. After incubation for 1 h at 37°C, wells were washed 3 times with SFM to remove extracellular parasites and cells fixed at different times post-infection in 4% formaldehyde.

### Chemicals

Caspase-1 inhibitor VI (YVAD) and caspase inhibitor VI (pan-caspase, Z-VAD) were from Calbiochem (Merck Chemicals, France). Macrophages were incubated with 50 µM of Caspase-1 inhibitor or 100 µM of pan-caspase inhibitor for 2 h before infection and during all the infection.

### Fluorescence microscopy

Infected macrophages were permeabilized with 0.002% saponine or 0.1% triton-×100 to detect SAG1 (mAb Tg05-54), GRA5 (mAb Tg17-113) or GRA3 (mAb Tg2H1). The rabbit anti-Tg small ubiquitin-like modifier (TgSUMO) polyconal antibody was kindly provided by M.A. Hakimi (Grenoble, France) [15]. To detect acidic vacuoles, infected macrophages were stained with 50 nM LysoTracker Red for 30 min prior to fixation. Cell death was analyzed by visualizing the uptake of Propidium Iodide (PI) (Molecular Probes). Alexa488 and Alexa594 antibody conjugates (Molecular Probes) were used as secondary antibodies. Coverslips were mounted in mowiol and observed and counted with a Zeiss Axioplan 2 microscope equipped for epifluorescence and phase-contrast. Kinetic experiments were performed on the IX2 Olympus microscope and analyzed with ScanR software.

### Caspase-1 activation assay

For analysis of caspase-1 activation, we used a fluorescent caspase-1 activity assay, FLICA, from Immunochemistry Technologies (Bloomington, MN). The assay was performed in 24-well plates, with 5.10^5^ cells per well. Cells were incubated with *T. gondii* and stained with FLICA reagent (FAM-YVAD-FMK) as recommended by the manufacturer. Fluorescence was measured on the IX2 Olympus microscope and analyzed with ScanR software.

### Annexin V assay

The level of apoptosis of infected LEW macrophages was assessed with Annexin V-propidium iodide staining. Rat peritoneal exudent cells were treated with etoposide (Sigma Aldrich, 50 µM) or incubated with parasites in SFM at a MOI of 1∶3 at 37°C, 5% CO_2_. After 1 h, cells were collected by centrifugation and incubated 4 h at 37° after addition of fresh medium. Macrophages were stained by the addition of Annexin V (Santa Cruz biotechnologies, FL-319) and Alexa488 secondary antibody (Molecular Probes) for 30 min and 1 µg/mL of PI for 5 min. Data acquisition was performed by flow cytometry on 4-colours FACSCalibur (BD Biosciences) equipped with 488 nm argon laser and CellQuest Software.

### DNA fragmentation analysis

Rat peritoneal macrophages (∼10^6^) were infected with 3×10^6^ parasites for 1 h, washed and returned to 37°C for 6 h, 12 h or 18 h. For positive control of apoptosis, macrophages were treated with 50 µM etoposide (Sigma Aldrich). Macrophages were treated in lysis-buffer (100 mM Tris, pH 8.0, 100 mM EDTA, 4% Sodium dodecyl sulfate (SDS) with 1 µg/ml RNAse (Roche) (30 min, 37°C) followed by treatment with 100 µg/ml proteinase K (Euromedex) (30 min, 55°C). After extraction with phenol/chloroform, DNA was recovered by precipitation and analysed on 1.8% agarose gels.

### Radical oxygen species production by macrophages

Rat peritoneal exudent cells were incubated with parasites in SFM at a MOI of 1∶3 at 37°C, 5% CO_2_ for 15 min or 4 h and then incubated with 0.45 µM of dihydro-rhodamine 123 (DHR, Sigma Aldrich) 15 min at 37°C. At the end of incubation, FACS lysing buffer (BD Bioscience, Pont de Claix, France) was added to each sample and incubated for 15 min. The samples were then washed in PBS before analysis with a FACSCalibur flow cytometer (Becton Dickinson) and the CellQuest Pro software (Becton Dickinson).

### Immunoblotting

Cells were lysed in cold RIPA (50 mM Tris-Hcl pH 7,4, 150 mM NaCl, 1% NP40, 0,25% Na-deoxycholate) buffer supplemented with protease inhibitors and centrifuged at 4°C and 13,000 *g* for 10 min. Supernatant of infected or uninfected macrophages (5.10^5^ cells/well) were collected and precipitated with TCA (trihloroacetate) for 10 min at 4°C prior centrifugation at 16 000 g for 5 min. Pellets were washed two times in acetone then dried and resuspended in laemmli buffer. Protein extracts were subjected to electrophoresis on a 12% Tris-HCl SDS-PAGE and transferred to PVDF membranes (Amersham). Membranes were blocked for 1 h in TTBS (100 mM Tris-HCl, 0.9% NaCl, and 0.05% Tween 20) containing 5% skim milk before incubating overnight at 4°C with primary 1/500 anti-caspase 3 (Cell Signaling), 1/1000 anti-IL-1β (Millipore, AB1832P), 1/5000 anti-GAPDH (Santa Cruz) or 1/1000 anti-actin (Sigma Aldrich) antibodies followed by 1/10000 anti-rabbit secondary horseradish peroxidase (HRP)-linked antibodies (Jackson Immunoresearch). Visualization of signals was enhanced by luminol-based chemiluminescence (ECL, ThermoFisher Scientific).

### *T. gondii* proliferation assay in peritoneal macrophages

The intracellular growth of *T. gondii* in rat peritoneal macrophages was monitored by selective incorporation of [^3^H]uracil as previously described [32]. Briefly, 5×10^5^ macrophages were infected with 1.5×10^6^ parasites for 1 h in SFM at 37°C and 5% CO_2_. After washing to eliminate extracellular parasites, cells were cultured for 20 h in the presence of [^3^H]uracil (5 µCi per well, Ci = 37 GBq). Monolayers were washed three times in PBS, disrupted with 500 µl of lysis/scintillation solution (Optiphase Supermix, Perkin Elmer) and radioactivity measured by liquid scintillation counting using a Wallac MicroBeta TriLux (Perkin Elmer).

### Genetic markers and genotype analysis

Preparation of genomic DNA and genotyping were performed as described [33]. To genotype the 35 (LOU X BN) F2 rat progenies, six microsatellite markers (D10Rat49, D10Arb2, D10GF41, D10Rat27, D10Mgh4, D10Rat2) were selected to cover chromosome 10 with an average spacing of 20 Mb. For haplotype analysis, the polymorphism of 41 microsatellite sequences around the *Toxo1* locus were analysed in the nine strains. Among these 41 markers, 17 newly identified microsatellite markers (Table S1) were used in addition to the 24 microsatellite markers selected from Rat Genome Database (RGD).

### ELISA

The anti-*Toxoplasma* IgG response was measured by specific enzyme-linked immunosorbent assay (ELISA). Total *Toxoplasma* antigens were prepared as previously described [34]. Immuno plates Maxisorp (Nunc) were coated overnight at 4°C with *Toxoplasma* antigens at 20 µg/ml. After washing, saturation was 1 h at 37°C with PBS containing 5% of skim milk. Sera were diluted at 1/20 and 1/1000 in PBS-0.01% Tween 20 and incubated 1h30 at 37°C. Plates were then washed with PBS-0.01% Tween 20, and peroxydase-conjugated anti-rat IgG (KPL) secondary antibody diluted at 1/5000 was incubated 1 h at 37°C. Finally, after six washes, 100 µl of substrate TMB-hydrogen peroxyde (TMB Ultra 1 Step, ThermoScientific) solution was added to the wells. The color reaction was stopped adding 50 µl of 3 N HCl. Optical densities at 492 nm and 630 nm were measured using an *ELx800* absorbance microplate reader (Bio TeK Instruments). Results were expressed as arbitrary units.

### Cyst detection

Each rat brain was removed and homogenized in 16 ml of PBS. Brain suspensions were clarified by gentle incubation in proteinase K buffer (proteinase K 0.4 µg/ml, 10 mM Tris pH 8, EDTA 1 mM, sodium dodecyl sulfate 0.2%, sodium chloride 40 mM) for 15 min at 56°C. The reaction was stopped by incubation with PMSF 2 mM for 5 min at room temperature. Then, the suspension was washed with PBS and resuspended with FITC-*Dolichos biflorus* agglutinin (Vector laboratories, CA USA) 20 µg/ml for 30 min, at room temperature. After washing in PBS, rat brains were resuspended in 6 ml of PBS, distributed into six-well culture plates (1 ml per well) and cysts counted visually with an Axiovert 40 CFL inverted fluorescence microscope (Zeiss) [35].

### *Toxo1* sequencing

A custom-made SureSelect oligonucleotide probe library was designed by IntegraGen (Evry, France) to capture the chr10 region containing *Toxo1* locus (chr10: 57,200,000–58,200,000). The eArray web-based probe design tool was used for this purpose (https://earray.chem.agilent.com/earray). A total of 56,450 probes, covering a target of 6830692 bp, were synthesized by Agilent Technologies (Santa Clara, CA, USA). Library preparation, capture enrichment, sequencing, and variants detection and annotation, were performed by IntegraGen (Evry, France). Briefly, 3 µg of each genomic DNA were fragmented by sonication and purified to yield fragments of 150–200 bp. Paired-end adaptor oligonucleotides from Illumina were ligated on repaired DNA fragments, which were then purified and enriched by six PCR cycles. 500 ng of the purified libraries were hybridized to the SureSelect oligo probe capture library for 24 h. After hybridization, washing, and elution, the eluted fraction underwent 14 cycles of PCRamplification. This was followed by purification and quantification by qPCR to obtain sufficient DNA template for downstream applications. Each eluted-enriched DNA sample was then sequenced on an Illumina GAIIx as paired-end 75 bp reads. Image analysis and base calling was performed using Illumina Real Time Analysis (RTA) Pipeline version 1.10 with default parameters. Sequence reads were aligned to the reference rat genome (UCSC rn4) using commercially available software (CASAVA1.7, Illumina) and the ELANDv2 alignment algorithm. Sequence variation annotation was performed using the IntegraGen in-house pipeline, which consisted of gene annotation (RefSeq), detection of known polymorphisms (dbSNP 125) followed by mutation characterization (exonic, intronic, silent, nonsense etc.).

### Statistical analyzes

For *in vivo* experiments, data are expressed as means ± SEM and the significance of differences found between groups was initially derived from a Kruskal-Wallis H test and subsequently confirmed by the Mann-Whitney test. For *in vitro* experiments, data are expressed as means ± SD and the significance of differences found between groups was determined using two-tailed Student's *t* test.

## Supporting Information

Figure S1 **The refractoriness of LEW macrophages acts against type I and type II parasites.** (**A**) The intracellular growth of type I (RH) (n = 3) and type II (Prugniaud) (n = 3) *T. gondii* within permissive macrophages from LEW.BNc10-F congenic lines and non-permissive macrophages from LEW was measured by monitoring [^3^H] uracil incorporation into Toxoplasma cells. Results were normalized according to the values obtained in BN macrophages. (**B**) The Type I (n = 5) and type II (n = 3) *T. gondii*-induced cell death of LEW.BNc10-F and LEW macrophages was monitored by PI uptake. Columns and bars show mean ± SD; _*_, *p*<0.05.
(TIF)

Figure S2 **Genetic variability in coding and non-coding sequences of** ***Toxo1*** **locus (A and B) and a chromosome 9 locus (C and D).** Classical multidimensional scaling of the *Toxo1* mutation data matrix. Numeric values on the x and y coordinates are non-absolutes. Red circles: Resistant strains; Blue circles: Susceptible strains; Violet circles: Overlapping between resistant and susceptible strains.
(TIF)

Figure S3 **PARP is lacking in rat peritoneal macrophages.** PARP and its cleaved form was revealed by western blotting of lysates from LEW BMDM (bone marrow derived macrophages) untreated or treated 2 h with 1 µM of LPS, LEW infected (6 h) and uninfected peritoneal macrophages, and THP1 cell line as control.
(TIF)

Table S1 **Primers used for genotyping.**
(DOCX)

Table S2 **Comparison of** ***Toxo1*** **gene expression between permissive (BN) and non-permissive (BN.LEWc10-Cg) peritoneal macrophages.** The expression levels were normalized to *Hprt* expression and the indicated values specify the differential fold expression (calculated by ΔΔCt method) between permissive (BN) and non-permissive (BN.LEWc10-Cg) peritoneal macrophages.
(DOCX)

Text S1 **Supporting Materials and Methods.**
(DOCX)
