# Supplementary figures and images for: A Highly Conserved Toxo1 Haplotype Directs Resistance to Toxoplasmosis and Its Associated Caspase-1 Dependent Killing of Parasite and Host Macrophage
Source: PLoS Pathog. 2014 Apr 3;10(4):e1004005. doi: 10.1371/journal.ppat.1004005 (PMC3974857; doi:10.1371/journal.ppat.1004005)

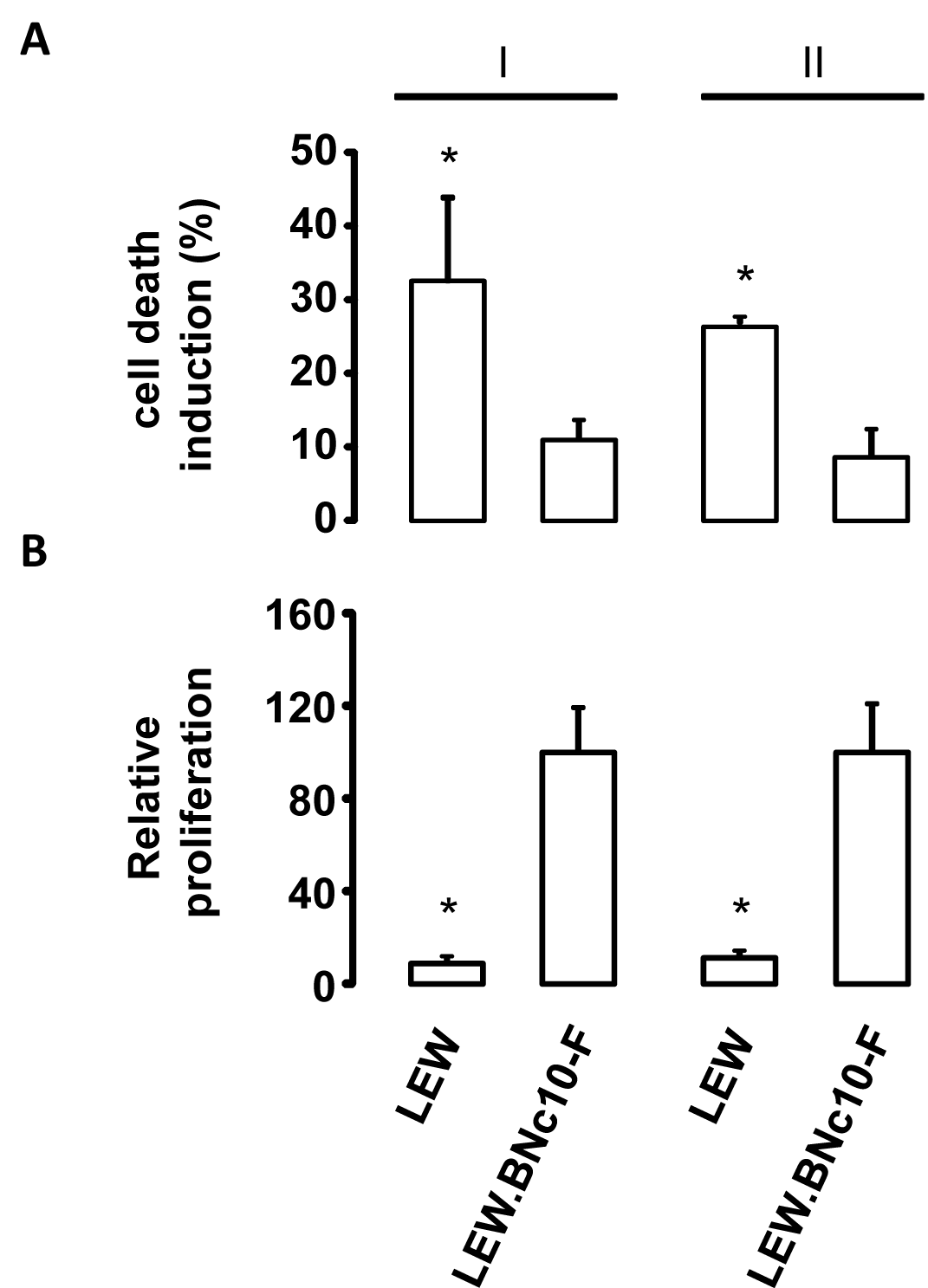

Supplement: Figure S1 — The refractoriness of LEW macrophages acts against type I and type II parasites. (A) The intracellular growth of type I (RH) (n = 3) and type II (Prugniaud) (n = 3) T. gondii within permissive macrophages from LEW.BNc10-F congenic lines and non-permissive macrophages from LEW was measured by monitoring [3H] uracil incorporation into Toxoplasma cells. Results were normalized according to the values obtained in BN macrophages. (B) The Type I (n = 5) and type II (n = 3) T. gondii-induced cell death of LEW.BNc10-F and LEW macrophages was monitored by PI uptake. Columns and bars show mean ± SD; *, p<0.05. (TIF) [file ppat.1004005.s001.tif]

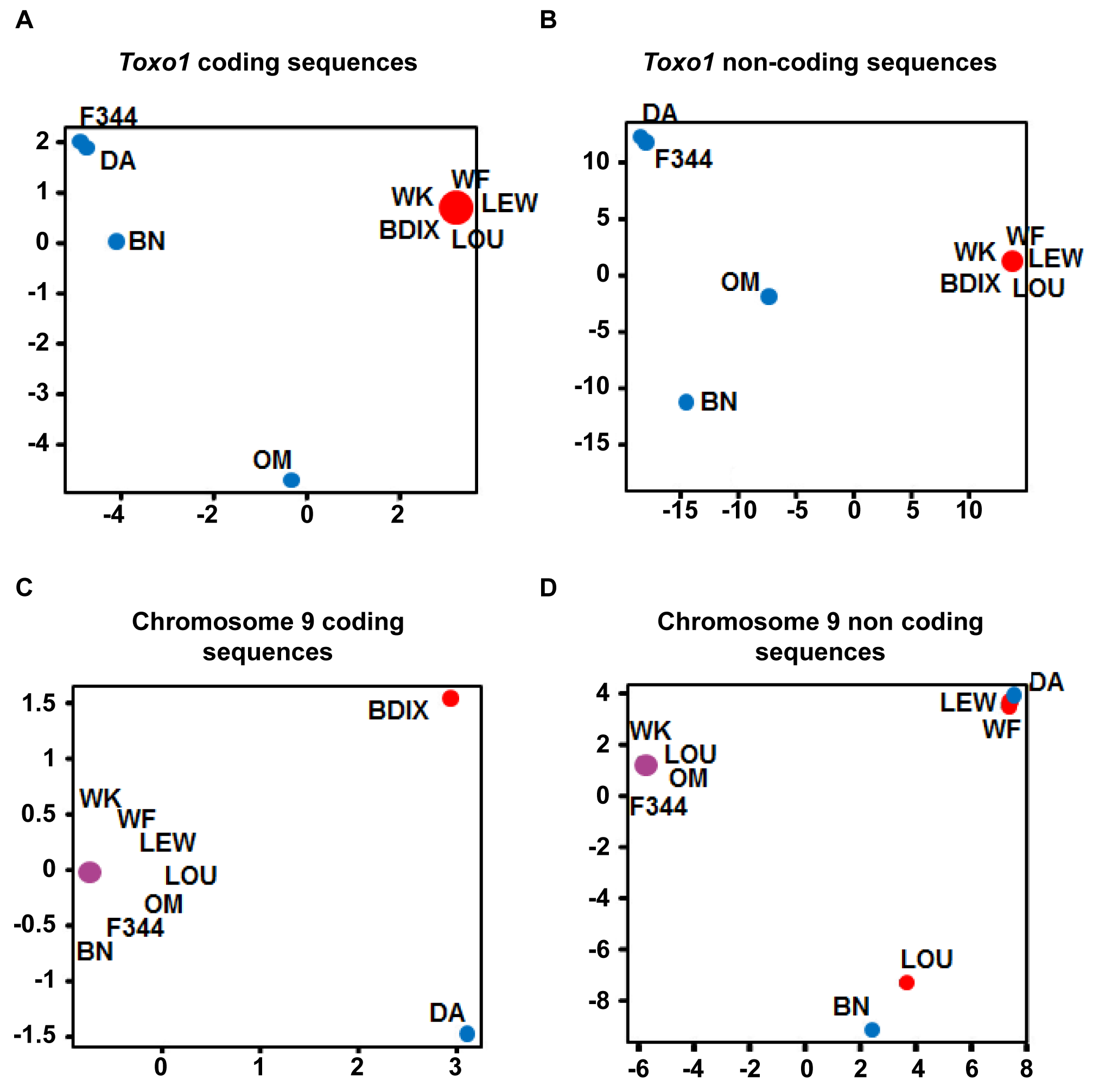

Supplement: Figure S2 — Genetic variability in coding and non-coding sequences of Toxo1 locus (A and B) and a chromosome 9 locus (C and D). Classical multidimensional scaling of the Toxo1 mutation data matrix. Numeric values on the x and y coordinates are non-absolutes. Red circles: Resistant strains; Blue circles: Susceptible strains; Violet circles: Overlapping between resistant and susceptible strains. (TIF) [file ppat.1004005.s002.tif]

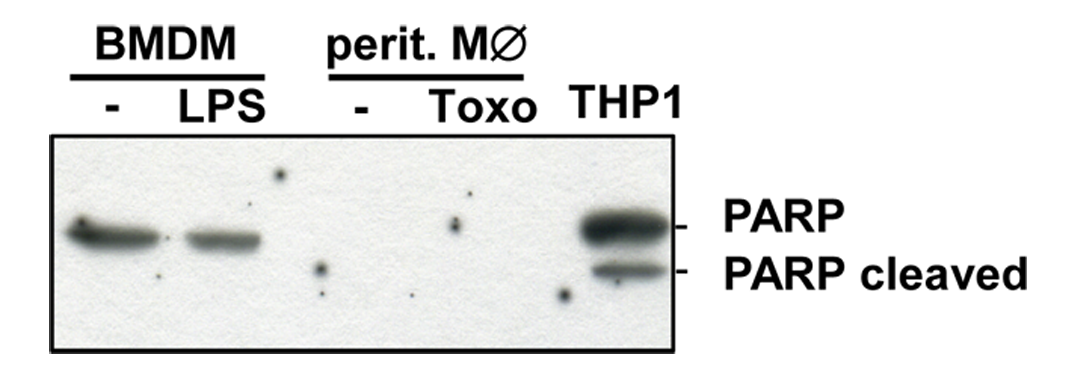

Supplement: Figure S3 — PARP is lacking in rat peritoneal macrophages. PARP and its cleaved form was revealed by western blotting of lysates from LEW BMDM (bone marrow derived macrophages) untreated or treated 2 h with 1 µM of LPS, LEW infected (6 h) and uninfected peritoneal macrophages, and THP1 cell line as control. (TIF) [file ppat.1004005.s003.tif]
