# Supplementary material for: A Highly Conserved Toxo1 Haplotype Directs Resistance to Toxoplasmosis and Its Associated Caspase-1 Dependent Killing of Parasite and Host Macrophage
Source: PLoS Pathog. 2014 Apr 3;10(4):e1004005. doi: 10.1371/journal.ppat.1004005 (PMC3974857; doi:10.1371/journal.ppat.1004005)
Supplement: Table S1 — Primers used for genotyping. (DOCX) [file ppat.1004005.s004.docx]

**Table S1.** Primers used for genotyping

| **markers** | **Forward primers** | **Reverse primers** |
| --- | --- | --- |
| **D10GF27** | ctaggaagcagccaagcatc | tggattaggtggaccacagc |
| **D10GF28** | tgcaggaatagaaaccctga | ccaagggggtgtgtacaagt |
| **D10GF39** | cctatgcctcacacacatgc | gcttttggcctcagctttt |
| **D10GF41** | ccagctccacatgatttcaa | taattggctggggacaagag |
| **D10GF101** | acatttcttcctggcagggtacac | cagttaggtggtagaggccaaagg |
| **D10GF57** | agctcctcatgttgtggtga | ctcctggtgtgcatgaagaa |
| **D10GF60** | agagttcattcagggcatgg | ctcatggctggcctctattc |
| **D10GF52** | cttccattctcctgccactc | ctaaggtctgggggtcctgt |
| **D10GF30** | aaatccccaaccctgatttt | tccccagaacacacatggta |
| **D10GF33** | cgtgttaggcaagcacactc | tggcacacatactcctgcac |
| **D10GF34** | tgaaagaggaggcaaggaga | tgtattcctagcccccaacc |
| **D10GF1** | aatgtggtgtcaggatgacc | gcatcagagaaaatactggaagg |
| **D10GF2** | aaggaggcataggcacacat | cgtctgtctgtctgtctctgtg |
| **D10GF3** | cccaacccaagaaacaaaaa | ttgcctctacctcccagatg |
| **D10GF5** | gaccacagggagaggagaaa | ccatactgggtgggcagat |
| **D10GF44** | ttcaactcctctgaattggaac | tgtgtatggaggaccattcg |
| **D10GF14** | tgcatgtgtgtgtgtgtgtg | tttggtcatggtgtttggtc |
