# Supplementary material for: A Highly Conserved Toxo1 Haplotype Directs Resistance to Toxoplasmosis and Its Associated Caspase-1 Dependent Killing of Parasite and Host Macrophage
Source: PLoS Pathog. 2014 Apr 3;10(4):e1004005. doi: 10.1371/journal.ppat.1004005 (PMC3974857; doi:10.1371/journal.ppat.1004005)
Supplement: Table S2 — Comparison of Toxo1 gene expression between permissive (BN) and non-permissive (BN.LEWc10-Cg) peritoneal macrophages. The expression levels were normalized to Hprt expression and the indicated values specify the differential fold expression (calculated by ΔΔCt method) between permissive (BN) and non-permissive (BN.LEWc10-Cg) peritoneal macrophages. (DOCX) [file ppat.1004005.s005.docx]

**Table S2.** Comparison of *Toxo1* gene expression between permissive and non-permissive macrophages

| **Genes** | **Ratio refractory/sensitive** |
| --- | --- |
| ***Med11*** | ND |
| ***Cxcl16*** | 0.5 ± 0.1 |
| ***Zmynd15*** | ND |
| ***Tm4sf4*** | ND |
| ***Vmo1*** | ND |
| ***Gltpd2*** | 1.2 ± 0.5 |
| ***Psb6*** | 1.2 ± 0.2 |
| ***Pld2*** | 0.6 ± 0.3 |
| ***Mink1*** | 2.8 ± 1.6 |
| ***Chrne*** | 0.9 ± 0.1 |
| ***Gp1ba*** | undetectable |
| ***Slc25a11*** | 1 ± 0.3 |
| ***Rfn167*** | undetectable |
| ***Pfn1*** | 0.9 ± 0.2 |
| ***Eno3*** | 0.9 ± 0.3 |
| ***Spag7*** | ND |
| ***Camta2*** | 0.7 ± 0.1 |
| ***Inca1*** | undetectable |
| ***Kif1C*** | 0.9 ± 0.2 |
| ***Zfp3*** | ND |
| ***Rabe1*** | 1.2 ± 0.2 |
| ***Nup88*** | 0.9 ± 0.3 |
| ***Rpain*** | 0.5 ± 0.1 |
| ***C1qbp*** | 0.8 ± 0.1 |
| ***Dhx33*** | 1.0 ± 0.6 |
| ***Derl12*** | 0.9 ± 0.2 |
| ***Mis12*** | ND |
| ***Nlrp1a*** | 1.7 ± 0.7 |
| ***Nlrp1b*** | undetectable |
