# Supplementary material for: A Highly Conserved Toxo1 Haplotype Directs Resistance to Toxoplasmosis and Its Associated Caspase-1 Dependent Killing of Parasite and Host Macrophage
Source: PLoS Pathog. 2014 Apr 3;10(4):e1004005. doi: 10.1371/journal.ppat.1004005 (PMC3974857; doi:10.1371/journal.ppat.1004005)
Supplement: Text S1 — Supporting Materials and Methods. (DOCX) [file ppat.1004005.s006.docx]

**Text S1. Supporting Materials and Methods.**

**Real time PCR**

To determine the expression of *Toxo1* genes total RNA from 2M of peritoneal macrophages was extracted with NucleoSpin RNAII kit (Macherey-Nagel). Reverse transcription was performed on 1µg of RNA using SuperScript II reverse transcriptase (Life Technologies) and according to the manufacturer’s recommendations. The real-time PCR mix consisted of 4 μl of cDNA template, 0.6 μl of each primer (300nM), and 10 μl of MESA GREEN qPCR Mastermix Plus (Eurogentec), with the final volume made up to 20 μl with RNase- and DNase-free water. The cycling conditions included an initial denaturation for 5 min at 95°C, 40 cycles of 95°C for 30 s, 60°C for 30 s, and 72°C for 30 s, and a final melting curve. Cycling was performed using **StepOnePlus Real-Time PCR System** (Applied Biosystems).

**Multidimensional scaling**

Genetic variability across the nine strains was analyzed by a method implemented into the R statistical software [].(1, 2) A Classical multidimensional scaling (MDS) of the *Toxo1* mutation data matrix was performed: we applied a principal coordinate’s analysis (Gower, 1966; Cox and Cox, 1994). Principal Coordinates Analysis (as others MDS) is a method to explore and to visualize similarities or dissimilarities of data. It starts with a similarity matrix or dissimilarity matrix (= distance matrix) and assigns for each item a location in a low-dimensional space, e.g. as a 2D graphics.

**PARP immunoblotting**

Cells were lysed in cold RIPA (50mM Tris-Hcl pH 7,4, 150mM Nacl, 1% NP40, 0,25% Na-deoxycholate) buffer supplemented with protease inhibitors and centrifuged at 4°C and 13,000*g* for 10 min. Protein extracts (20 µg/lane) were subjected to electrophoresis on a 12% Tris-HCl SDS-PAGE and transferred to PVDF membranes (Amersham). Membranes were blocked for 1 h in TTBS (100 mM Tris-HCl, 0.9% NaCl, and 0.05% Tween 20) containing 5% skim milk before to be probed overnight at 4°C with primary 1/500 anti-PARP (Cell Signaling) antibody followed by 1/10000 anti-rabbit secondary horseradish peroxidase (HRP)-linked antibodies (Jackson Immunoresearch). Visualization of signal was enhanced by luminol-based chemiluminescence (ECL, ThermoFisher Scientific).

**References**

**1. Ihaka R and Gentleman R (1996) R: A Language for Data Analysis and Graphics*.* J Comput Graph Stat 5: 299-314.**

**2. Team RDC (2003) R: A Language and Environment for Statistical Computing*.* R Foundation for statistical Computing 1: 1999-2002**
